# Advances in plant essential oils and drug delivery systems for skincare

**DOI:** 10.3389/fphar.2025.1578280

**Published:** 2025-04-17

**Authors:** Wang Yihan, Dou Jinjin, Wang Yingqi, Mu Guanai, Zhang Xiwu

**Affiliations:** ^1^ Institute of Chinese Medicine, Heilongjiang University of Traditional Chinese Medicine, Harbin, Heilongjiang, China; ^2^ The Four Hospital of Heilongjiang University of Traditional Chinese Medicine, Harbin, Heilongjiang, China

**Keywords:** phytotherapy, essential oil, skin care, delivery system, intrinsic efficacy

## Abstract

**Background:**

Essential oils, often referred to as “liquid gold,” are renowned for their broad biological activity. Ancient Egyptians used essential oils’ antibacterial and antiseptic effects to preserve mummies, ancient Greeks used olive oil for sun protection, and ancient Chinese used essential oils to treat wounds. When essential oils are applied to the facial skin, their potent anti-inflammatory, antioxidant, and antibacterial pharmacological characteristics provide various benefits, including sunscreen, skin-whitening, and anti-aging effects.

**Purpose:**

This paper aims to summarize the application of plant essential oil in skin whitening, anti-inflammatory, antioxidant and antibacterial in recent years, and deeply analyzes the internal relationship between essential oil and modern drug delivery system, expounds how to overcome the limitations of essential oil through specific drug delivery system, to enhance its biological activity and stability, realize sustained release and reduce its potential toxicity, and also discusses the positive effects of essential oil on brain function through olfactory pathway, emphasizes the possible safety risks in the use of essential oil, and puts forward corresponding suggestions for use.

**Methods:**

Using keywords such as “essential oils,” “antioxidant,” “anti-tyrosinase,” Antibacterial Effects and anti-inflammatory,” “anti-anxiety,” and “drug carrier delivery systems,” a comprehensive search was conducted in the PubMed, CNKI, Baidu, and Wanfang databases to summarize articles from the past 5 years. Further screening was performed to select studies demonstrating the efficacy of essential oils through topical or external application.

**Results:**

Various essential oils showed their efficacy as strong oxidants, antibacterial agents, anti-inflammatory agents, and skin-whitening agents. Combined with a new drug delivery system, it not only enhances the biological activity of essential oil but also reduces the inherent defects of essential oil, such as volatility, irritation, and toxicity, and has a targeted delivery effect. At the same time, the integration of essential oil into skin care products can make use of the dual functions of smell and the epidermal system to nourish and repair the skin and maximize the pharmacological effects of essential oil.

**Conclusion:**

This review delves into the application of essential oils and delivery systems, advocating for a broader integration of natural plant resources with modern technology. By strategically utilizing essential oils, we can promote the sustainable development of the global economy. However, extensive clinical trials are still required to evaluate the effectiveness and safety of essential oil delivery systems.

## 1 Introduction and historical uses of essential oils in skincare

Essential oils (EOs) are concentrated plant extracts obtained through processes such as steam distillation (Kapadia et al., 2022). They are typically extracted from the leaves, flowers, skins, roots, seeds, pericarps, and other parts of aromatic plants and consist of a variety of volatile chemical metabolites, such as terpenes, esters, alcohols, and aldehydes (Bolouri et al., 2022). Owing to their diverse biological activities, environmental friendliness, and ease of acquisition, EOs have long been used in various traditional medicine approaches (Khalil et al., 2021).

EO applications date back thousands of years to ancient civilizations such as Egypt, Arabia, Greece, and China (Long et al., 2022). In ancient Egypt, EOs with antibacterial and preservative properties, such as *Boswellia sacra Flück.*, Commiphora myrrha (T.Nees) Engl., and Cinnamon (Cinnamomum verum J. Presl), were used to embalm bodies during mummification (Vora et al., 2024). Similarly, Lavender (Lavandula angustifolia Mill.) was used in traditional medicine across Asia, Europe, ancient Greece, and Rome, and is mentioned in both the Bible and ancient Jewish texts for its anti-inflammatory and antibacterial effects (Hancianu et al., 2013). The ancient Romans applied oils, including EOs from Clove (Syzygium aromaticum (L.) Merr. and L.M.Perry) and Lavender, on their skin for fragrance, whereas the Greeks used olive oil as sunscreen (Liu and Zhang, 2017). Monoï oil, widely used among the Tahitian tribes in Polynesia for skincare, is primarily applied to hydrate the skin and safeguard it against sun exposure (McMullen and Dell’Acqua, 2023). In 1919, French chemist René-Maurice Gattefossé accidentally burned his hand during a laboratory explosion. Subsequently, he used Lavender EO to treat the infected wound and discovered its remarkable healing effects. This discovery has prompted researchers to conduct in-depth research on the medicinal value of EOs and promote the wide application of EOs in medical and skin care fields.

This paper summarizes the latest research findings and academic perspectives on the applications of plant EOs in skin whitening, anti-inflammatory, antioxidant, and antibacterial treatments, along with a brief introduction to the structure and function of the skin. It provides an in-depth analysis of the intrinsic relationship between EOs and drug delivery systems, elucidating how specific drug delivery systems can overcome the limitations of EOs. Additionally, the paper discusses the positive effects of EOs on brain function through the olfactory pathway and highlights potential safety risks associated with their use, offering corresponding recommendations. These insights provide valuable references for further research.

## 2 Constitution of the skin

**FIGURE 1:**
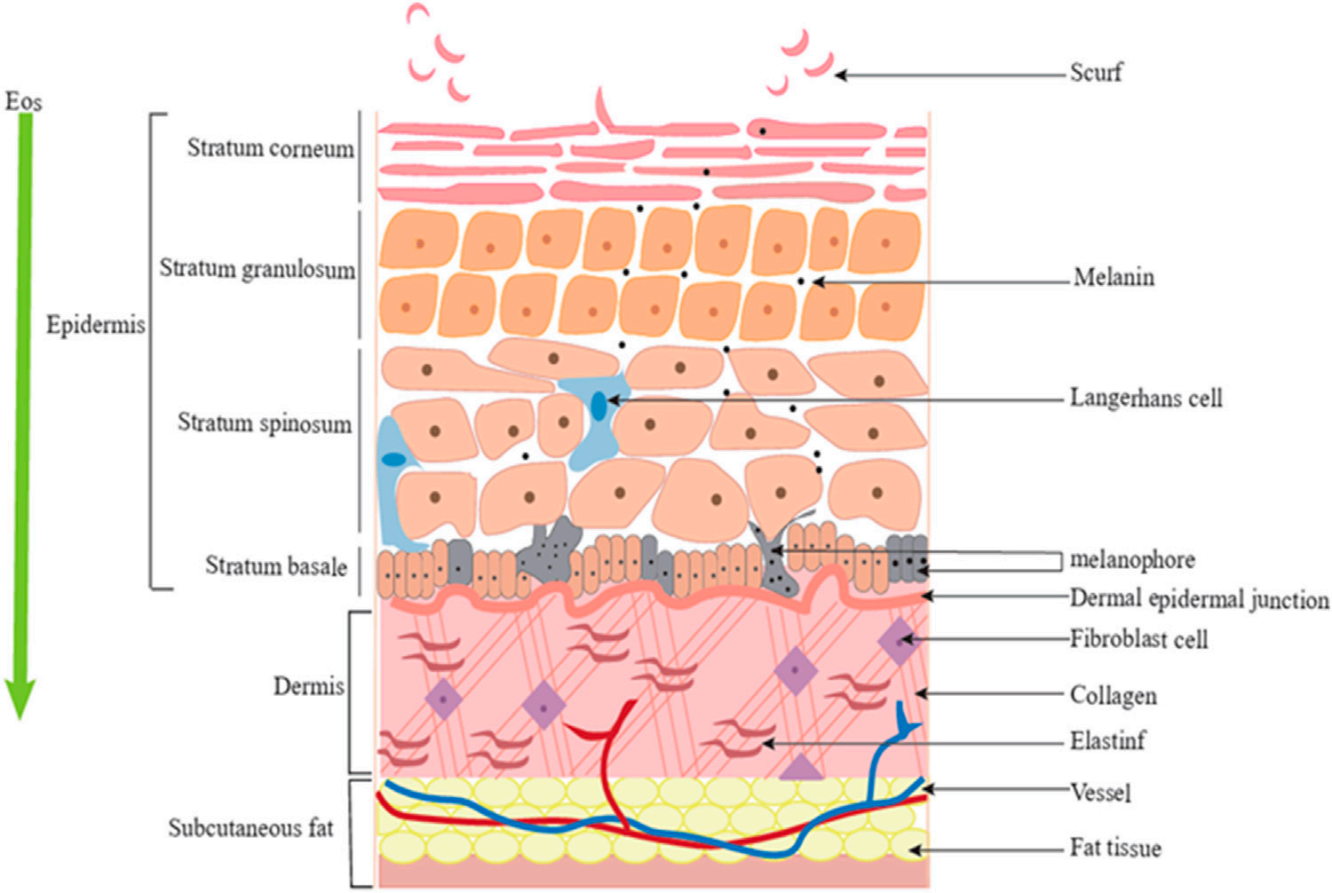
Schematic diagram of facial skin composition.

The skin serves as the body’s first line of protection against harmful external stimuli and performs several crucial functions. First, it prevents excessive water loss, thereby preserving the body’s hydration balance (Afshari et al., 2024). Secondly, the skin acts as an effective barrier against toxins and infections. As shown in Figure 1, facial skin primarily comprises the epidermis, dermis, and subcutaneous tissue (Nascimento et al., 2024). Keratinocytes within the epidermis play a key role in forming the skin’s protective barrier and help distribute melanin produced by melanocytes. Melanocytes in the basal layer of the epidermis are responsible for melanin synthesis and influence skin color. EOs can interact with stratum corneum proteins, causing conformational changes that disrupt the lipid phase, thereby weakening the skin barrier. This process promotes the transdermal delivery of both hydrophobic and hydrophilic metabolites, enhances the deep penetration of active metabolites, and improves the anti-aging and whitening properties of products (McMullen and Dell’Acqua, 2023; Yang et al., 2024a). Below the epidermis lies the dermis, which is rich in collagen and elastic fibers that provide skin elasticity and firmness, maintaining a youthful appearance. The subcutaneous layer, comprising fatty tissue, lymphatic vessels, nerves, and blood vessels, provides nourishment and support. Moreover, the skin plays a pivotal role in metabolic processes, material absorption, and protection against biological, physical, and chemical threats (Michalak et al., 2021). In summary, the skin is not only a protective barrier but also an organ essential for maintaining overall physical health and beauty.

## 3 Pharmacological activities of essential oils

### 3.1 Whitening effect of essential oils

**FIGURE 2:**
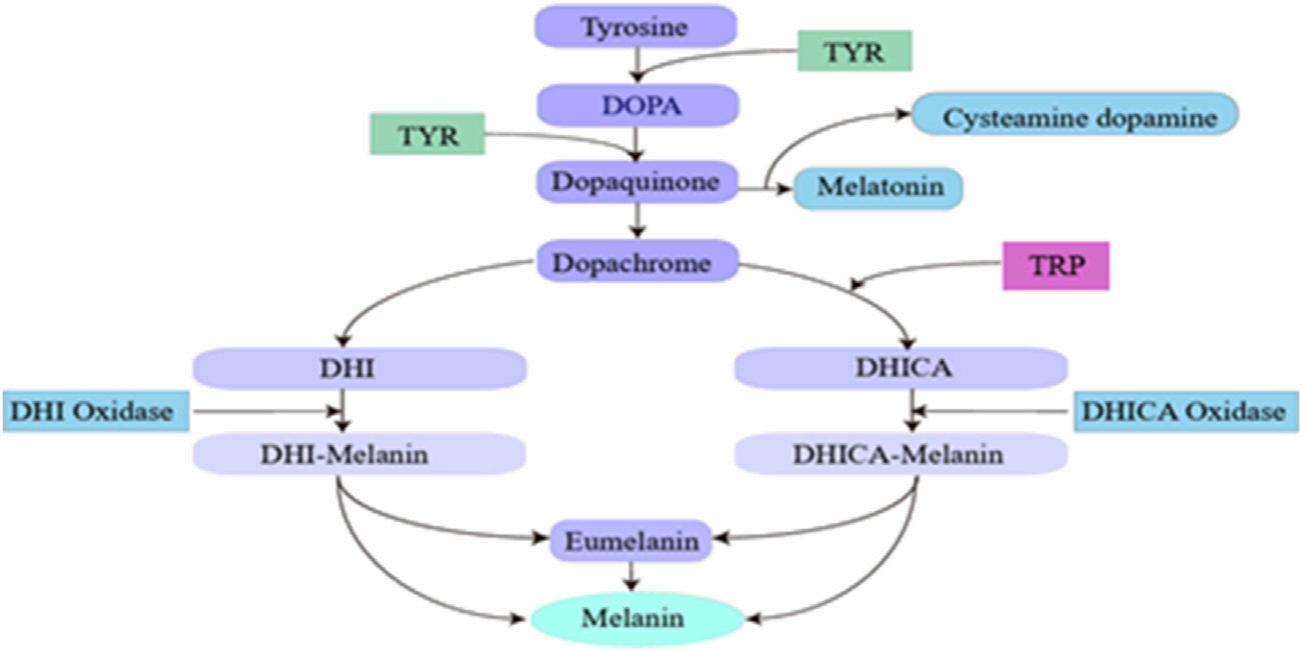
Flow chart of melanin formation.

Melanin, the primary pigment in human hair, eyes, and skin, is synthesized in melanosomes within melanocytes (Yu et al., 2024). Tyrosinase (TYR) is a vital copper-containing metalloenzyme crucial for melanin synthesis in mammals (Ma and Lu, 2020). In melanocytes, tyrosine undergoes a series of oxidation processes mediated by TYR and other enzymes. Specifically, tyrosinase-related protein (TRP) converts dopachrome into 5,6-dihydroxy indole-2-carboxylic acid (DHICA), while TRP-1 facilitates the conversion of DHICA to indole-5,6-quinone-2-carboxylic acid, ultimately resulting in melanin production. The microphthalmia-associated transcription factor (MITF) plays a pivotal role in regulating the expression of TYR, TRP-1, and TRP-2, thereby significantly impacting melanin synthesis. Furthermore, MITF is activated through Mitogen-Activated Protein Kinase (MAPK) signaling cascades, which stimulate melanogenesis in response to diverse external stimuli (Kim et al., 2021; Yang et al., 2023a). This process is summarized in Figure 2. The synthesized melanin is subsequently released from melanocytes into the intercellular space as pigment particles and gradually transferred to the cytoplasm of surrounding keratinocytes, ultimately reaching the stratum corneum. While an appropriate amount of melanin protects the skin from detrimental environmental factors (He et al., 2023), an excess can lead to pigmentation issues, a dull skin tone, and spot development (Nurani et al., 2023).

Kojic acid, arbutin, ascorbic acid, and hydroquinone (Draelos et al., 2020; Mahalapbutr et al., 2023; Zahoor et al., 2023) are well-known chemical inhibitors of TYR. However, their use can pose potential health risks. Since 2001, hydroquinone has been excluded from skincare products due to cancer risks associated with continuous exposure (Zolghadri et al., 2023). Kojic acid, while effective for skin whitening, is limited by its carcinogenic potential and instability during storage (Liu et al., 2024a). When arbutin is applied to the skin, microbes or ultraviolet radiation (UVR) can convert it into hydroquinone (Boo, 2021). Conversely, plants are increasingly valued in skin-whitening cosmetics, with up to 3/5 of products in medical skincare deriving from plant-specific metabolic metabolites (Capetti et al., 2021). Plant extracts and metabolites have demonstrated efficacy in preventing excessive melanin formation in the epidermis (Kanlayavattanakul and Lourith, 2018). For instance, *in vitro* experiments utilizing EO from *Echinophora chrysantha* Freyn and Sint. Have demonstrated an inhibitory effect on TYR activity, subsequently affecting melanin synthesis and reducing its content (Kanbolat et al., 2024). *In vivo* experiments on zebrafish have demonstrated that *Boswellia papyrifera* (Caill. Ex Delile) Hochst. EO can effectively inhibit melanin production, with no significant toxic effects observed within the tested concentration range (Yan et al., 2024). Table 1 summarizes plant EOs that exert skin-whitening effects by inhibiting the expression of melanin, TYR, or TRP through *in vivo* or *in vitro* experiments.

**TABLE 1:** Skin whitening effect of different EOs.

EOs	Plant material	Major constituent	Method	Effect	References
Aralia elata (Miq.) Seem	Flower	γ-tocopherol; cyclotetracosane	*In vitro*, α-MSH stimulates B16BL6	B16BL6 cells were treated with EOs at a concentration of 200 μg/mL, the anti-melanogenesis-related responses reached their maximum (inhibition of MITF, TYR, TRP-1, and TRP-2 expression, and downregulation of MAPKs phosphorylation)	Kim et al. (2024)
Etlingera elatior (Jack) R.M.Sm	Leaf	a-pinene; humulene	*In vitro*, melanoma cell lines (A375 and B16F10)	The IC_50_ for reducing melanin content in A375 and B16F10 cell lines was 252.12 ± 3.02 and 253.56 ± 3.65 μg/mL, which is approximately 2.8-fold lower than kojic acid	Sangthong et al. (2022)
Hypericum empetrifolium Willd	Above ground; Root	alloaromodendrene; α-pinene	*In vitro*, TYR inhibition test	The EO exhibits moderate anti- TYR activity at a concentration of 200 μg/mL compared to the standard compound	Boga et al. (2021)
Litsea cubeba (Lour.) Pers	Fruit	geranial (α-citral); neral (β-citral)	*In vitro*, TYR inhibition test; B16-F10 melanoma cell line	EO inhibits TYR activity; at a concentration of 80 μg/mL, the viability of B16-F10 cells decreases to its lowest level	Qiu et al. (2022)
*Alpinia nantoensis* F.Y.Lu and Y.W.Kuo	Leaf; Rhizome	α-pinene and _D_-limonene	*In vitro*, 20 μM FSK stimulates B16-F10	At a concentration of 100 μg/mL, the EO exhibited stronger inhibition of melanogenesis compared to arbutin or kojic acid; Suppression of MITF activity	Kumar et al. (2020)
Mentha × piperita L	Leaf	Menthol; D-limonene	*In vitro*, TYR inhibition test	suppression of TYR expression	Pavlić et al. (2021)
Dalbergia pinnata (Lour.) Prain	Leaf	Elemicin; Methyl eugenol	*In vitro*, TYR inhibition test; *In vivo*, zebrafish embryos test	At a concentration of 0.02 mg/mL, the EO achieved a TYR inhibition rate of 74%, whereas arbutin at the same concentration showed an inhibition rate of approximately 51%; at 30 mg/L, the EO demonstrated more pronounced anti-melanogenic effects compared to arbutin	Zhou et al. (2020)
*Origanum vulgare L*	Above ground	β-caryophyllene epoxide	*In vitro*, TYR inhibition test	The TYR inhibitory activity of the EO is 26.5% ± 0.3%	Moghrovyan et al. (2019)
*Melaleuca quinquenervia* (Cav.) S.T.Blake	Leaf	1,8-cineole α-pinene; viridiflorol	*In vitro*, α-MSH stimulates B16	suppression expression of melanin and TYR	Chao et al. (2017)
Juniperus phoenicea L	leaves; berries	α-pinene	*In vitro*, α-MSH stimulates B16	At a concentration of 20 μg/mL, the EO significantly reduces TYR activity	Mansour et al. (2023)
Sigesbeckia glabrescens (Makino) Makino	Flower	lauric acid; methyl undecanoate	*In vitro*, α-MSH stimulates B16BL6	At a concentration of 20 μg/mL, the EO reaches its peak inhibition of TYR activity (126.75% ± 0.60%); suppression expression of MITF, TYR, TRP-1, and TRP-2	Lee et al. (2023)
Agathis dammara (Lamb.) Rich. and A. Rich	Leaf	δ-cadinene; γ-gurjunene	*In vitro*; TYR inhibition test; *In vivo*, zebrafish embryos test	At a concentration of 43.48 μg/mL, the EO reduces melanin formation in zebrafish embryos by 50%, outperforming kojic acid	Ho et al. (2023)
*Impatiens textori* Miq	Flower	Palmitoleic acid; palmitelaidic acid	*In vitro*, α-MSH stimulates B16BL6	The concentration of EO was 200 μg/mL, which significantly inhibited TYR activity; suppression of TYR, MITF, and melanin	Won et al. (2022)
*Calocedrus formosana* (Florin) Florin	Wood	α-terpineol; shonanic acid	*In vitro*, α-MSH (100 μM) and FSK (20 µM) stimulates B16-F10	At a concentration of 80 μg/mL, the melanin inhibition effect of the EO is comparable to that of kojic acid at 200 μg/ML; The activity of TYR and the expression of TRP-1, TRP-2, and MITF were significantly inhibited	Hsiao et al. (2021)
Camellia japonica L	Seed	Hexamethy-lcyclotrisiloxane	*In vitro*, α-MSH stimulates B16-F10	suppression of TRP-1, TRP-2, and melanin	Ha et al. (2021)

IC_50_, half-maximal inhibitory concentration; TD_50_, half-maximal toxicity dose; μM, micromolar; B16-F10, malignant melanoma cell line of C57BL/6 J mice; A375, human melanoma cell line; B16BL6, melanoma-associated cell line; α-MSH, Alpha-Melanocyte-Stimulating Hormone; B16, melanoma-associated cell line.

### 3.2 Anti-aging effect of essential oils

The aging of facial skin is a progressive process, resulting from the interplay between internal aging factors and external influences (Carvalho et al., 2023; Liu et al., 2024c). Signs such as increased wrinkles, reduced firmness and elasticity, dry keratinization, and excessive pigment deposition indicate a loss of skin suppleness due to aging (Banov et al., 2023). Prevailing theories on skin aging highlight the roles of free radicals (Harman, 1956) and photoaging (Fisher et al., 1996). The antioxidant activity of EOs efficiently counteracts free radicals, thereby slowing the aging process (Maache et al., 2023). EOs also absorb UV rays, reducing skin damage and promoting the repair and regeneration of skin cells.

#### 3.2.1 Free radical theory

**FIGURE 3:**
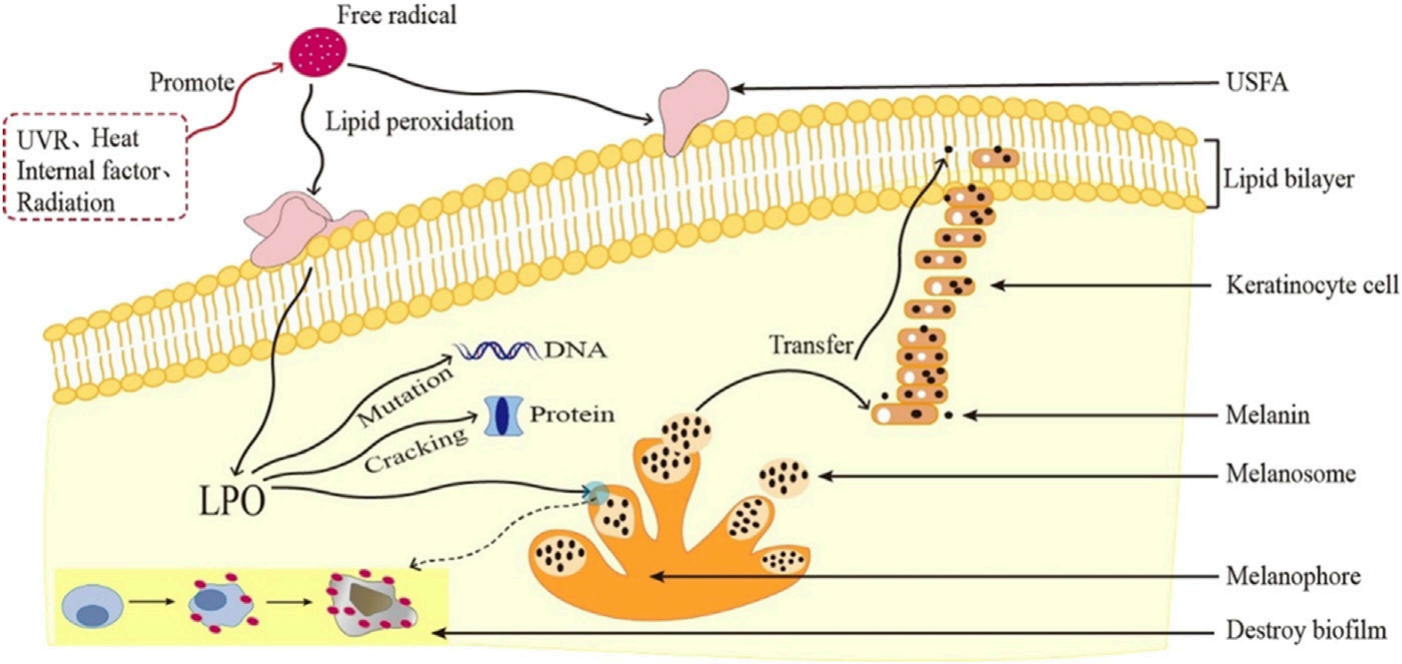
Schematic diagram of aging caused by free radicals.

Harman introduced the free radical aging hypothesis in 1954, suggesting that both internal and external oxidants and free radicals damage cells, altering their structure and function (Harman, 1956). External factors, such as light, radiation, and heavy metals (Chen et al., 2023a; Xie et al., 2020), as well as normal cell metabolism, produce free radicals. Increased free radical levels accelerate aging and contribute to aging-related diseases (Tamagno et al., 2022). Normally, the body’s antioxidant system mitigates free radicals to prevent their accumulation (Di Meo and Venditti, 2020). However, with age, the body’s defenses weaken, leading to an abnormal accumulation of free radicals, particularly reactive oxygen species (ROS). Excess ROS reacts with unsaturated fats, creating lipid peroxides (LPO) through lipid peroxidation (Zhou et al., 2024), thereby damaging cellular membranes and organelles. This process contributes to cell damage, necrosis, aging, and an increased risk of disease. High free radical levels can also cause protein breakdown, DNA mutations, and potentially cancer (Bakheit et al., 2024) (Figure 3).

Synthetic antioxidants, such as butylated hydroxyanisole and 2,6-di-tert-butyl-p-cresol, are widely utilized in the food and skincare sectors (Xu et al., 2021). However, research suggests that they may increase the risk of cancer (Riaz et al., 2023). Consequently, EOs have garnered remarkable interest due to their superior antioxidant properties and safety profile. They contain various antioxidant metabolites that effectively eliminate excess free radicals, donate electrons or hydrogen, and inhibit lipid peroxidation, thereby preventing skin damage and aging. Notably, the anti-aging effects of EOs are achieved by inhibiting free radical-driven signal transduction (Guo et al., 2021). Table 2 summarizes EOs with antioxidant properties and briefly presents the relevant experimental results.

**TABLE 2:** Antioxidant effects of different EOs.

Eos	Plant material	Major constituent	Method	Effect	References
*Thymus pulegioides* L	Above ground	Thymol; Carvacrol	*In vitro*, DPPH	At a concentration of 1 mg/mL, its activity is stronger than that of vitamin E	Dong et al. (2023)
*Thymus thracicus* Velen		Thymol			
*Thymus serpyllum* L		Oxygenated monoterpenes			
Salvia acerifolia B.L. Turner	Above-ground part of full flowering	1,8-cineole; camphor	*In vitro*, ABTS; Detection of ROS and Antioxidant Enzymes’ Activity	Suppression ROS/stimulates GST, CAT	Badalamenti et al. (2023), Henríquez et al. (2023)
Origanum aciculare (Waldst. and Kit.) Kuntze	Leaf	Carvacrol; p-cymene; Eucalyptol	*In vitro*, DPPH	Radical scavenging rate of EO is higher than that of BHT	Zejli et al. (2023)
*Pistacia lentiscus* L	Fruit	Limonene; α-pinene	*In vitro*, DPPH, ABTS, FRAP	The antioxidant activity range is 29.64 ± 3.04 to 73.80 ± 3.96 μg/mL	El Omari et al. (2023)
*Curcuma alismatifolia* Gagnep	Rhizome	Camphor; Curzerenone	*In vitro*, DPPH, ABTS	The IC_50_ in DPPH tests is 19.1 ± 0.16 μg/mL; the IC_50_ in ABTS tests is 15.24 ± 0.17 μg/mL	Mohanty et al. (2023)
*Curcuma aromatica* Salisb		Xanthorrhizol; Curlone (β-Turmerone)		The IC_50_ in DPPH tests is 23.1 ± 0.18 μg/mL; the IC_50_ in ABTS tests is17.4 ± 0.18 μg/mL	
Curcuma aeruginosa Roxb		γ-Gurjunene; (Z)-Caryophyllene		The IC_50_ in the DPPH tests is 36.8 ± 0.14 μg/mL; the IC_50_ in the ABTS test is 29.4 ± 0.17 μg/mL	
*Curcuma longa* L	Leaf	α-phellandrene; 2-carene	*In vitro*, DPPH, ABTS	IC_50_ = 10.04 ± 0.03 μg/mL for DPPH assays; IC_50_ = 14.12 ± 0.21 μg/mL for ABTS	Visakh et al. (2023)
Cinnamomum malabatrum (Burm.f.) J. Presl	Leaf	linalool; caryophyllene	*In vitro*, DPPH, ABTS, FRAP	The IC_50_ in the DPPH test is 21.50 ± 0.17 μg/mL; the IC_50_ in the ABTS tests is 36.91 ± 0.41 μg/mL	Kuttithodi et al. (2023)
Ocimum americanum L	Leaf	Camphor; limonene	*In vitro*, DPPH; ABTS; FRAP; metal-chelating techniques	DPPH radical IC_50_13.42 ± 1.03 μg/mL); ABTS activity of 2085.07 ± 7.43 µM TEAC/g oil	Mahendran and Vimolmangkang (2023)
Ocimum basilicum L		3,7-dimethyl-, (Z)-(-citral) Estragole		DPPH radical IC_50_11.56 ± 0.89 μg/mL; ABTS activity of 2,842.12 ± 10.39 µM TEAC/g oil	
*Laurus nobilis* L	Leaf	1,8-cineole sabinene; linalool	*In vitro*, DPPH	Antioxidant activity 76.84%	Ailli et al. (2023)
*Pistacia lentiscus* L		3-methylpentylangelate		Antioxidant activity 71.53%	
*Cedrus atlantica* (Endl.) Manetti ex Carrière	Wood	β-himachalene		Antioxidant activity 62.38%	
Mentha × piperita L	Leaf; Flower	Carvone; Limonene	*In vitro*, DPPH	DPPH radical IC_50_ 15.93 mg/mL	Zekri et al. (2023)
Kaempferia galanga L	Rhizomes	trans-ethyl p-methoxycinnamate	*In vitro*, DPPH, ABTS, Hydroxyl radical scavenging activity, Reducing power assay; *In vivo*, zebrafish embryos test	Attenuated MDA, ROS generation, cell death, and lipid peroxidation/increase in SOD, CAT, GSH-Px	Wang et al. (2023b)
Rosmarinus officinalis L	Branch	Camphor; verbenone	*In vitro*, DPPH, ABTS	Suppression of ROS	Huang et al. (2023)
Lavandula angustifolia Mill	Flower	linalool; borneol	*In vitro*, DPPH	EOs in Capracotta areas DPPH radical IC_50_ 26.26 mg/mL	Caprari, et al. (2023)
Zingiber officinale Roscoe	Rhizomes	zingiberene; (+)-β-cedrene	*In vitro*, DPPH	Free radical clearance rate of ∼95% at a concentration of 0.25%	Zhang et al. (2022a)
Artemisia annua L	Leaf; Stem	Artemisinone; (+)-α-pinene	*In vitro*, DPPH, Hydroxyl radical test	DPPH clearance rate is 40.03%, and the hydroxyl radical clearance rate is 92.97%	Panyod et al. (2024)
Cinnamomum bodinieri H. Lév	Leaf	Citral; Neral; geranial	*In vitro*, DPPH, ABTS, FRAP	DPPH IC_50_ = 6.887 ± 0.151 mg/mL; ABTS IC_50_ = 19.08 ± 0.02 mg/mL	Ling et al. (2022)
*Epilobium angustifolium* L	Leaf	α-caryophyllene oxide; eucalyptol	*In vitro*, DPPH	2.445 ± 0.025 mg Trolox/g EOEa in DPPH	Nowak et al. (2021)
Ferulago abbreviata C.C.Towns	Fruit	(Z)-β-ocimene; α-phellandrene	*In vitro,* DPPH	DPPH EC_50_ = 68.75 μg/mL	Narimani et al. (2022)
*Aloysia citriodora* Palau	Leaf	geranial; rans-1,2-Bis-(1-methyl phenyl); cyclobutene	*In vitro*, DPPH	DPPH IC_50_ = 11.74 ± 0.18 μg/mL	Jaradat et al. (2021)
Eucalyptus grandis W. Hill	Leaf	1,8-cineole; α-pinene	*In vitro*, DPPH, ABTS	DPPH IC_50_ = 42.5 mg/mL; ABTS IC_50_ = 7.4 mg/mL	Zhou et al. (2021)
Syzygium aromaticum (L.) Merr. and L.M.Perry	Flower	Eugenol	*In vitro*, DPPH	DPPH EC_50_ = 0.36 μL/mL	Vella et al. (2020)
*Dalbergia pinnata* (Lour.) Prain	Leaf	Elemicin; Methyl eugenol	*In vitro*, DPPH, ABTS	DPPH IC_50_ = 0.038 mg/mL; ABTS IC_50_ = 0.032 mg/mL	Zhou et al. (2020)
Anthemis palestina (Reut. Ex Kotschy) Reut. Ex Boiss	Above ground	γ-muurolene; €-β-farnesene	*In vitro*, DPPH	DPPH IC_50_ = (1.00 ± 0.03) ×10^−2^ μg/mL	Al-Qudah et al. (2023)
Boswellia carteri Birdw	Resin	Limonene; α -phellandrene	*In vitro*, DPPH	DPPH clearance rate is 86.44% ± 2.12%	Obiș;tioiu et al. (2023)
Tetraclinis aphylla (L.) Rothm	leafy branches	Camphor; Bornylacetate; Borneol	*In vitro*, DPPH, FRAP	DPPH IC_50_ = 266.9 ± 5.4 μg/mL; FRAP EC_50_ = 433.16 ± 4.13 μg/mL	Asbabou et al. (2024)

CAT, antioxidant enzyme catalase; GST, glutathione-S-transferase; SOD, superoxide dismutase; EC_50_, median effective concentration; MDA, malondialdehyde; GSH-Px, glutathione peroxidase; FRAP, ferric ion reducing antioxidant power; DPPH:2,2-diphenyl-1-picrylhydrazyl; ABTS:2,2′-azino-bis(3-ethylbenzothiazoline-6-sulfonic acid.

#### 3.2.2 Photoaging theory

The skin, which covers approximately 1.5–2 m^2^ of the human body, serves as its primary physiological barrier (Xu et al., 2023) and encounters external stimuli such as UVR, smoke, and harmful pollutants (Ge et al., 2024). UVR, particularly UVA (wavelength range 320–400 nm) and UVB (wavelength range 290–320 nm) (Gęgotek et al., 2017), is a major exogenous factor contributing to skin aging (Yang et al., 2023b). Prolonged UVR exposure can lead to hyperpigmentation, skin laxity, severe wrinkling, and an increased risk of skin cancer (Kim et al., 2023; Liu et al., 2024b). UVR also triggers skin inflammation, increasing ROS levels that activate signaling in keratinocytes and fibroblasts, thereby upregulating matrix metalloproteinases (MMPs) (Sim et al., 2023). These proteolytic enzymes degrade collagen, elastin, and other connective tissue proteins, disrupt the extracellular matrix, and inhibit new collagen synthesis (Apte and Parks, 2015). Specifically, UVR exposure enhances the activity of MMPs—such as collagenase, 92-kd gelatinase, and stromelysin—in the skin (Birkedal-Hansen, 1987). As a result, these enzymes accelerate the breakdown of endogenous type I collagen fibers by up to 58%, thereby accelerating the skin aging process. Moreover, adverse skin reactions from the overuse of chemicals in sunscreens are becoming more common (Messias et al., 2023). Therefore, identifying natural and effective sunscreen metabolites is critical. EOs have shown significant potential in sun protection due to their ability to absorb UV radiation while scavenging free radicals, ultimately reducing photodamage and delaying skin aging. The EOs listed in Table 3 are renowned for their sun protection efficacy and demonstrate significant potential for natural skincare applications.

**TABLE 3:** Sunscreen characteristics of different EOs.

EOs/oil	Major constituent	Method	Effect	References
*Boswellia papyrifera* (Caill. Ex Delile) Hochst	n-octyl acetate; α-pinene	*In vivo*, Hairless rats irradiated by UVB	Inhibition of MAPK (pERK, pJNK, and pp38) and MMPs (MMP1 and MMP9)/Increase the expression of TGF-β and procollagen I synthesis	Kotb et al. (2023)
Zingiber montanum (J. Koenig) Link ex A.Dietr	Sabinene; erpinene-4-ol	*In vitro*, UVB-irradiated HDFn	Inhibition of MMP/increased expression of type I procollagen synthesis	Navabhatra et al. (2022)
Oncosiphon *suffruticosum* (L.) Källersjö	Camphor; filifolone	*In vitro*, SPF *via* UV spectroscopy	SPF 2.299	Adewinogo et al. (2021)
*Coriandrum sativum* L	linalool	*In vitro*, Screening of enzyme activities related to skin aging; *in vivo*, anti-wrinkle activity	Inhibition of the activity of collagenase, elastase, TYR, and hyaluronidase activity, as well as the levels of MDA, COX-2, PGE-2, MMP-1, JNK, and AP-1/Increase the expression of TGFβ, TGFβII, and SMAD3 protein levels	Salem et al. (2022)
*Artemisia sieversiana* Ehrh	Artemisia ketone; Artemisol	*In vivo*, UVB-irradiated mice	Increased expression of SOD/Inhibition of MDA, MMP-1 and MMP-3, as well as epidermal thickness, inflammatory cell infiltration, collagen degradation, and elastin aberrance	Zhou et al. (2022)
Magnolia sieboldii K. Koch	β-elemene; γ-terpinene	*In vivo*, UVB irradiated mice	Inhibition of skin photoaging, TNF-α, IL-6, IL-10	Zhang et al. (2021)
Blumea balsamifera (L.) DC.	caryophyllene; borneol			
Calendula americana Mill	α-Cadinol	*In vitro*, SPF *via* UV spectroscopy	SPF 8.36	Lohani et al. (2019)
Geranium acaule L	Citronellol; geraniol		SPF 6.45	
Syzygium cumini (L.) Skeels	α-pinene; β-pinene; €-β-caryophyllene	*In vitro*, anti-aging experiment	Inhibition of collagenase, elastase, and hyaluronidase activity	Ashmawy et al. (2023)
Kaempferia galanga L	ethyl cinnamate	*In vitro*, UVB-irradiated	Moderate sun protective activity and reduce nitric oxide production induced by LPS in macrophage cells	Chittasupho et al. (2022)

IL-6, Interleukin-6; IL-1β, Interleukin-1β; COX-2, Cyclooxygenase-2; PGE2, Prostaglandin E2; MMP-1, and MMP-3, Matrix Metalloproteinases-1,-3; TNF-α, Tumor Necrosis Factor-α; HaCaT, human keratinocyte cell line; HDFn, human dermal fibroblast cells; SPF, sun protection factor.

### 3.3 Antibacterial effects of essential oils

**FIGURE 4:**
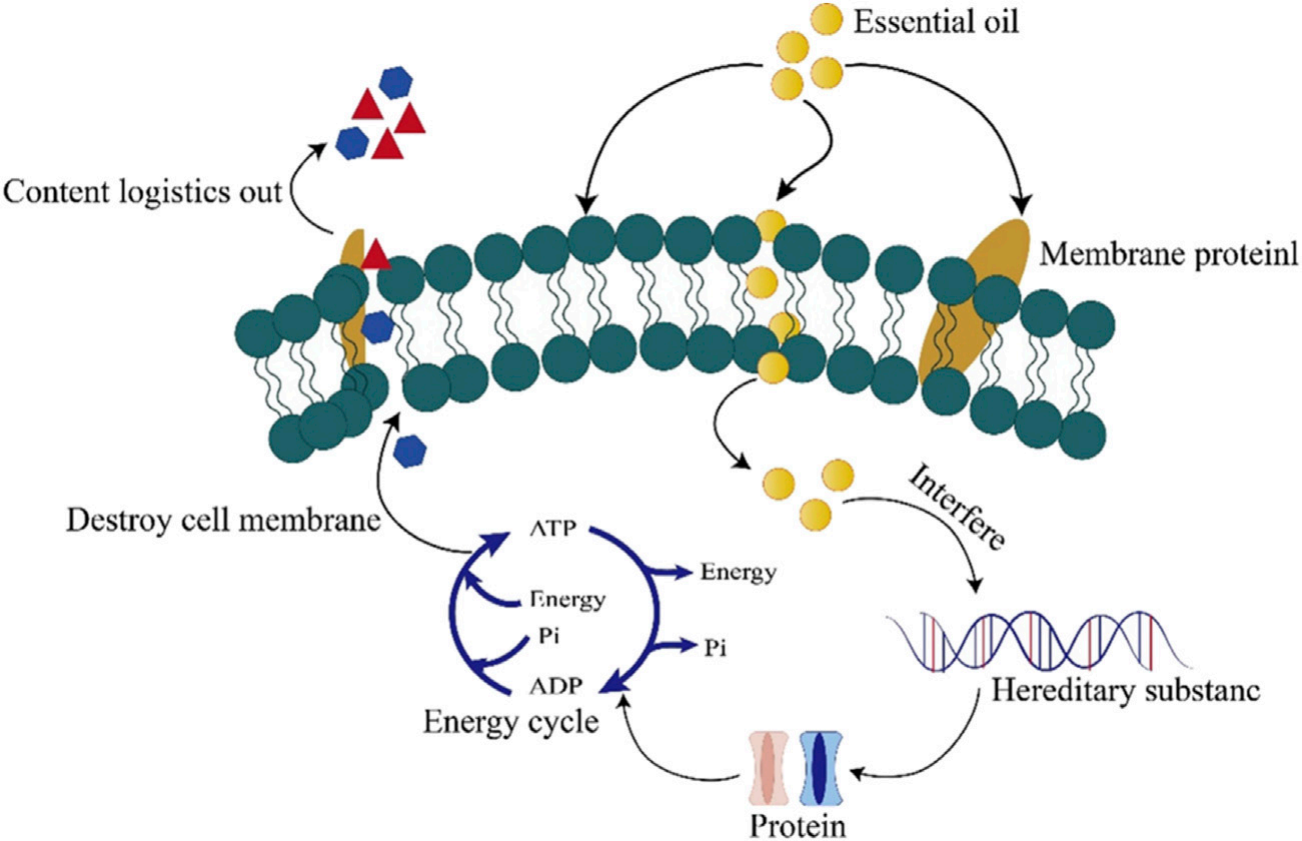
Schematic diagram of the antibacterial mechanism of EOs.

Skin is not only a physical barrier isolating the body from the outside world but also a habitat for various microorganisms (Dessinioti and Katsambas, 2024). An imbalance in the skin microbial community can lead to diseases such as acne, atopic dermatitis, and eczema. Many chemical preservatives (phenoxyethanol, methyl p-hydroxybenzoate [p-HBA], butyl p-hydroxybenzoate [BuP], chlorobenzyl ether, and sodium benzoate [SB]) are added to skincare products to prevent microbial contamination during production, storage, and use, and to prolong shelf life (Alshehrei, 2024; Głaz et al., 2023). However, they cannot protect the skin from bacterial pollution and may be harmful to users. For instance, p-HBA can impact nervous system development in zebrafish (Merola et al., 2024) and reduce male fertility (Shen et al., 2023). BuP is hepatotoxic in aquatic animals (Yin et al., 2023), negatively affects heart development (Zhu et al., 2023), and inhibits neural crest cell proliferation in zebrafish larvae, causing craniofacial deformities (Li et al., 2023b). Additionally, high concentrations of SB may be toxic to insects (Asejeje et al., 2024). The accumulation of these chemical preservatives on the skin may promote aging and pigmentation. Excessive or improper use of chemical preservatives poses risks to human health and the environment (Nowak-Lange et al., 2022). With increasing awareness of health and environmental protection, the reliance on chemical preservatives in skincare is a growing concern (Jafarizadeh-Malmiri et al., 2022). Plant EOs, with their natural composition, favorable safety profile, and potent antibacterial properties, are frequently utilized as alternatives to chemical preservatives (Cunha et al., 2022). As lipophilic substances, EOs can disrupt lipids and proteins, increase membrane permeability, and cause leakage of cellular contents (Álvarez-Martínez et al., 2021) (Figure 4). For instance, *in vitro* studies show that *Lemon verbena* EOs can damage the cell walls of cultivated yellow croakers, increasing membrane permeability and causing leakage of cellular contents, ultimately leading to cell death (Gao et al., 2023). The antibacterial properties of plant EOs hold great potential for use as natural preservatives (Table 4 summarizes the related EOs).

**TABLE 4:** Antibacterial effects of different EOs.

EOs	Plant material	Major constituent	Method	Effect	References
*Salvia mirzayanii* Rech.f. and Esfand	Above ground	(α)-terpineol; linalyl acetate	*In vitro*, Antibacterial Effects experiment	Against *S. aureus*, EOs demonstrate an MIC of 1.65 μL/mL, an MBC of 6.25 μL/mL, and a ZOI of 30 mm; Against *E. coli*, EOs show an MIC of 3.12 μL/mL, an MBC of 50 μL/mL, and a ZOI of 14 mm	Shaverdi et al. (2024)
*Tanacetum annuum* L	Above ground	Chamazulene; Camphor	*In vitro*, Agar disk diffusion method	Inhibition of G^+^ bacterial growth	Belcadi et al. (2023)
*Cinnamomum cassia* (L.) J.Presl	Bark	cinnamaldehyde; (+)-isomenthol	*In vitro*, Bacteriostatic Circle	Inhibition of *E. coli*, *B. subtilis*, *S. aureus*, *C. albicans*, and *P. aeruginosa* growth	Zhao et al. (2023)
Origanum vulgare L	Leaves	carvacrol; thymol			
Eucalyptus globulus Labill	Leaves	Myrcene	In vitro, Antibacterial Effects experiment; MIC, MBC assays	Inhibition of *B. bronchiseptica*, *S. epidermidis*, and *S. aureus* growth	Nasir Shah et al. (2023)
Rosmarinus officinalis L	Leaves	α-pinene; 1,8-cineole	*In vitro*, paper disc diffusion	Inhibition of *S. typhi* and *S. aureus* growth	Hashemi et al. (2023)
*Tetraclinis articulata* (Vahl) Mast	Leaves	bornyl acetate; α-pinene	*In vitro*, disc-diffusion technique; MIC, MBC assays	Inhibition of G+, G-, *C. albicans* growth	El Hachlafi et al. (2024)
cardamom (Elettaria cardamomum (L.) Maton)	Seed	1,8-cineole	*In vitro*, agar disk diffusion method	Inhibition of *L. monocytogenes*, *S. aureus*, *E. coli*, and *S. typhimurium* growth	Sobhy et al. (2023)
*Eucalyptus citriodora* Hook	Leaves	monoterpenoid aldehyde citronellal	*In vitro*, disc diffusion method; Antifungal activity	*S. aureus* DIZ: 32 ± 1.3 mm;*C. albicans* MIC: 65.1 ± 1.6 μg/mL	Fayez et al. (2023)
Eucalyptus camaldulensis Dehnh		β-cymene; 1,8-cineole		*S. aureus* DIZ: 34 ± 0.8 mm; *C. albicans* MIC: 45.3 ± 1.7 μg/mL	
Eucalyptus ficifolia F.Muell		trans-β-ocimene; 1,8-cineole		*S. aureus* DIZ: 36 ± 1.0 mm;*C. albicans* MIC: 71.1 ± 3.6 μg/mL	
Geranium acaule L	Leaves	Citronellol; terpinene	*In vitro*, disc diffusion; zone inhibitory	Inhibition of *S. epidermidis* and *E. coli* growth	Tansaoui et al. (2023)
Artemisia rutifolia Stephan ex Spreng	Above ground	α-thujone; β-thujone	*In vitro*, Disc diffusion method	Inhibition of Gram-positive bacteria and fungi growth	Dylenova et al. (2023)
*Ocimum forskolei* Benth	flowering aerial parts	methyl eugenol; eugenol	*In vitro*, Well-Diffusion Method	Inhibition of *E. coli*, *P. aeruginosa*, and *S. aureus* growth	Bader et al. (2023)
Zingiber officinale Roscoe	Rhizomes	Limonene; camphene	*In vitro*, Antibacterial Effects experiment	Inhibition of *S. aureus*, *E. coli*, and *C. albicans* growth	Berechet et al. (2023)
*Crithmum maritimum* L	Above ground	γ-Terpinene; sabinene; Thymylmethyl oxide	*In vitro*, agar diffusion method; microdilution assay	Inhibition of *B. cereus*; *S. aureus*; *L. plantarum*; *E. coli* growth	Pedreiro et al. (2023)
*Cymbopogon citratus* (DC.) Stapf	Stem leaf	geranial; neral	*In vitro*, Antibacterial Effects experiment	Inhibition of *E. coli*, *S. aureus* growth	Gaspar et al. (2022)
Helichrysum italicum (Roth) G.Don	herb; Flower	α-pinene; nerol	*In vitro*, Antibacterial Effects experiment	Antibacterial activity: the EOs from inflorescences > herb EOs	Węglarz et al. (2022)
Matricaria chamomilla L	Flower	cis-ene-yne-dicycloether	*In vitro*, Broth microdilution test	Inhibition of *S. aureus*, *P. aeruginosa*, *E. coli* growth	Pastare et al. (2023)
Chrysanthemum morifolium Ramat	Flower; stems-leaves; roots	caryophyllene	*In vitro*, microdilution method	*S. aureus* MIC: 10 mg/mL; *P. acnes* MIC: 25 mg/mL	Liu et al. (2022b)
Alpinia galanga (L.) Willd	Flower	Farnesen; aceteugenol	*In vitro*, Antibacterial Effects experiment	DIZ: 8.79–14.32 mm, MIC: 3.13–6.25 mg/mL, MBC: 6.25–12.50 mg/mL	Tian et al. (2022)

ATP, adenosine triphosphate; ADP, adenosine diphosphate; G^+^, Gram-positive bacteria; G^−^, Gram-negative bacteria; MIC, minimum inhibitory concentration; MBC, minimum bactericidal concentration; *E. coli*, *Escherichia coli*; *B. subtilis*: *Bacillus subtilis*; *S*, *aureus*: *Staphylococcus aureus*; *C. albicans*: *Candida albicans*; *P*, *aeruginosa*: *Pseudomonas aeruginosa*; *B*, *bronchiseptica*: *Bordetella bronchiseptica*; *S*, *epidermidis*: *Staphylococcus epidermidis*; *L*, *monocytogenes*: *Listeria monocytogenes*; *S*, *typhimurium*: *Salmonella typhimurium*; *S*, *typhi*: *Salmonella typhi*; DIZ, diameter of inhibition zone; ZOI, zone of inhibition.

### 3.4 Anti-inflammatory effect of essential oils

**FIGURE 5:**
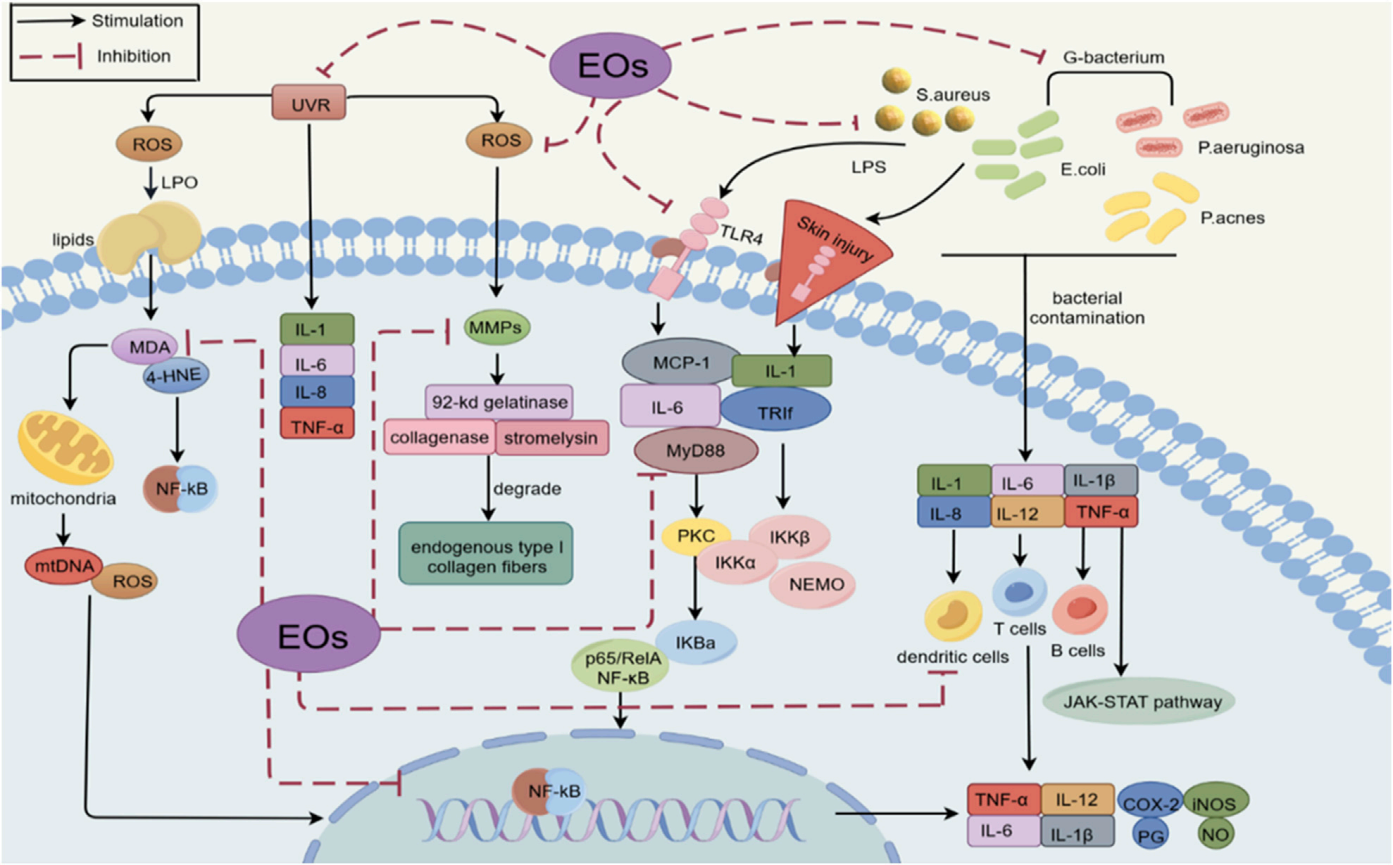
Schematic diagram of the anti-inflammatory effect of EOs (By Figdraw).

Inflammation is a complex defense mechanism that counteracts harmful external stimuli or abnormal internal signals. The immune system plays a central role in this process by eliminating the cause of disease and preserving the structural integrity of cells and tissues to maintain homeostasis. When the skin experiences irritation, bacterial infection, or damage, an inflammatory response is triggered. While moderate inflammation aids in repairing skin damage, excessive inflammation can lead to chronic skin diseases such as dermatitis, rosacea, and acne vulgaris. During this process, immune cells, including T cells, B cells, and dendritic cells, secrete inflammatory factors, most notably cytokines. Following skin damage, keratinocytes also become activated. The recognition and binding of specific pathogens or damaging factors to the pattern recognition receptor TLR (toll-like receptor)4 on the cell membrane triggers the release of inflammatory substances and subsequent immune cell activation (de Sousa et al., 2023; Jiang et al., 2020; Li et al., 2024a; Piipponen et al., 2020; Zhang et al., 2024a) (Figure 5). Current primary treatments for inflammation include corticosteroids, NSAIDs, and biologicals. However, drug resistance compromises their efficacy, necessitating novel therapeutic approaches (Russo et al., 2024). EOs exhibit potent anti-inflammatory properties by reducing capillary permeability and inhibiting both inflammatory cell activation and cytokine release. Thus, EOs are increasingly used as alternatives to traditional drugs for treating inflammation (Shen et al., 2017). For example, adapalene (ADA) is a commonly used gel for treating acne. When a tea tree EO nanoemulsion was combined with ADA, it produced a stronger therapeutic effect than ADA alone in clinical experiments, without increasing adverse effects (Najafi-Taher et al., 2022). Another clinical study revealed that adding 3% kānuka EOs to cream significantly improved eczema to a greater extent than the vitamin C control (Shortt et al., 2022). Table 5 summarizes EOs and their anti-inflammatory properties.

**TABLE 5:** Anti-inflammatory effects of different EOs.

EOs	Plant material	Major constituent	Method	Effect	References
Cinnamomum camphora (L.) J. Presl	Branch and leaf	linalool	*In vitro*, LPS-stimulated macrophages	Activates the Nrf2/HO-1 pathway and reduces TNF-α, IL-6, and IL-1β levels	Zhang et al. (2024d)
Prunus humilis Bunge	nucleolus	amygdalin	*In vivo*, DSS-induced ulcerative colitis mice	Inhibits the PI3K/AKT pathway to reduce the release of inflammatory factors	Wang et al. (2024b)
*Kaempferia galanga* (L.)	rhizomatous root	Curcumol; Curcumene	*In vivo*, GU rat model	Promotes the expression of PGE2, TGF-α, and EGF/inhibits NF-κB/COX-2 pathway, thus reducing IL-8 and TNF-α	Liu et al. (2023)
*Ferulago lutea* (Poir.) Grande	Umbellies of seeds	α-pinene; limonene	*In vitro*, LPS-stimulated macrophages	Decreases NO release as well as iNOS and pro-IL-1β protein levels	Alves-Silva et al. (2023)
Lavandula angustifolia Mill	flower	Linalool	*In vitro*, *P. aeruginosa* LPS-induced inflammation model	Inhibits the expression of IL-6, IL-8, IL-β, and TNF	Pandur, et al. (2021)
Salvia officinalis L	acrial part	12-O-methyl carnosol; eucalyptol	*In vitro*, COX-2 Inhibition Assayt	Inhibition of COX-2 expression; IC_50_ = 5.3 ± 0.62 g/mL	Alomar et al. (2023)
Cedrus atlantica (Endl.) Manetti ex Carrière	dry wood	β-himachalene; α-himachalene	*In vitro*, 5-LOX inhibition test	Inhibition of 5-LOX expression	El Hachlafi et al. (2023)
*Mesosphaerum suaveolens* (L.) Kuntze	Leaves	Β-caryophyllene; phyllocladene	*In vitro*, LPS-induced inflammation model	Inhibits the NF-κB pathway and reduces the mRNA of iNOS and COX-2, reducing the expression of IL-6, IL-1β, TNF-α, and ROS production	Mohanta et al. (2023)
Citrus medica L	skin	d-limonene; γ-terpinene	*In vivo*,ovalbumin-induced; *In vitro*, LPS-induced inflammation model	Suppresses the expression of IL-6, IL-1β, and TNF-α	Feng et al. (2023)
Rosa rugosa Thunb	flower	Citronellol; farnesol	*In vitro*, LPS-induced inflammation in RAW 264.7 cells	Inhibition of the NF-κB pathway and suppression of NO, ROS, and MDA	Raka et al. (2022)
Citrus maxima (Burm.) Merr	acrial part	D-limonene; Laurene	*In vitro*, LPS-induced inflammation model	Inhibits the expression of IL-6, TNF-α, ROS, IL-1, and COX-2	Nikolic et al. (2023), Zhang et al. (2022c)
Bursera graveolens (Kunth) Triana and Planch	Fruits	limonene	*In vivo*, Edema caused by AA	The thickness of mouse ear skin decreased by 25%/Inhibits the expression of IL-8, IL-17A, and IL-23	Sosa et al. (2023)
Dacryodes peruviana (Loes.) H.J. Lam	Fruits	α-phellandrene		The thickness of mouse ear skin decreased by 53.3%	
Mespilodaphne acuminata (Baker) Baill	Leaves	€-methyl cinnamate		The thickness of mouse ear skin decreased by 33.42%/Inhibits the expression of IL-17 A and IL-23	
Melaleuca armillaris (Sol. Ex Gaertn.) Sm	Leaves	1,8-cineole		The thickness of mouse ear skin decreased by 65.25%	
*Eugenia gracillima* Kiaersk	Leaves	D-germacrene; γ-muurolene-g	*In vivo*, Carrageenan-induced paw edema and peritonitis	Inhibition of inflammation, as well as leukocyte and neutrophil migration	Guedes et al. (2023)
Monarda didyma L	Above-ground part of flowering	Limonene; eucalyptol; β-pinene	*In vitro*, LPS-stimulated U937 cells	Inhibits TLR-4 signaling, reducing the expression of IL-6 and upregulating miR-146a	Fraternale et al. (2022)
Blumea lanceolaria (Roxb.) Druce	Leaves; Stem; Roots	o-cymene; carvacrol methyl ether	*In vitro*, RAW 264.7 cell model; *In vivo*, carrageenan-induced paw edema model	Inhibits the NF-κB pathway; the phosphorylation of IκBα; and the production of NO, TNF-α, IL-6, iNOS, and COX-2	Do et al. (2022)
Origanum compactum Benth	aerial part	Carvacrol; thymol; α-pinene	*In vitro*, 5-LOX inhibition test; *In vivo*, carrageenan-induced paw edema model	Inhibition of 5-LOX expression	Al-Mijalli et al. (2022)
Zingiber officinale Roscoe	Rhizomes	Zingiberene; (+)-β-cedrene	*In vivo*, TPA-induced ear swelling in mice	Inhibits the NF-κB pathway and decreases COX-2 and IL-6 expression	Zhang et al. (2022a)
*Hedychium flavum* Roxb	flower	β-pinene α-pinene	*In vitro*, LPS-induced release in RAW264.7; *In vivo*, xylene-induced ear edema model	Suppresses iNOS, COX-2, IL-6, TNF-α, and IL-1β expression as well as NO and PGE2 release *in vitro*/the levels of TNF-α, IL-6, and IL-1β were reduced *in vivo*	Tian et al. (2023)

DSS, dextran sulfate sodium; 5-LOX, lipoxygenase; NO, nitric oxide; iNOS, nitric oxide synthase; AA, Arachidonic Acid.

## 4 Challenges in essential oil application

In summary, EOs have unique biological activity and can be used in medicine, food, and skin care products. However, their high volatility and lipophilicity compromise stability during storage and use, particularly upon exposure to light, heat, O_2_, and UVR. Such exposure can diminish the active metabolites of EOs, thereby decreasing their efficacy. More critically, EOs may produce oxidation and isomerization products that are highly toxic and allergenic (Yi et al., 2022). For instance, the main component of anise, Clove, Cinnamomum verum J. Presl, and thyme EOs, trans-anethole, isomerizes into cis-anethole under ultraviolet light or high temperatures. Additionally, trans-anethole can be completely oxidized into benzaldehyde or isomerized into cis-anethole after being stored in light for 2 months at room temperature. Studies have shown that cis-anethole may cause stronger skin irritation, such as erythema, itching, and contact dermatitis, and increase the risk of allergic reactions (Castro et al., 2010). Additionally, a patch test conducted by Geier et al. on 12 EOs in 10,930 dermatitis patients revealed that 908 patients (8.3%) reacted to at least one EO. Among these, only six EOs (ylang-ylang, lemongrass, jasmine, sandalwood, Clove, and neroli) elicited positive patch test reactions in more than 1% of the patients (Geier et al., 2022). Although sensitization reactions to EOs do occur, they are generally uncommon. Nonetheless, safety concerns in practical applications should not be overlooked.

## 5 Essential oil^’^s drug delivery system

**FIGURE 6:**
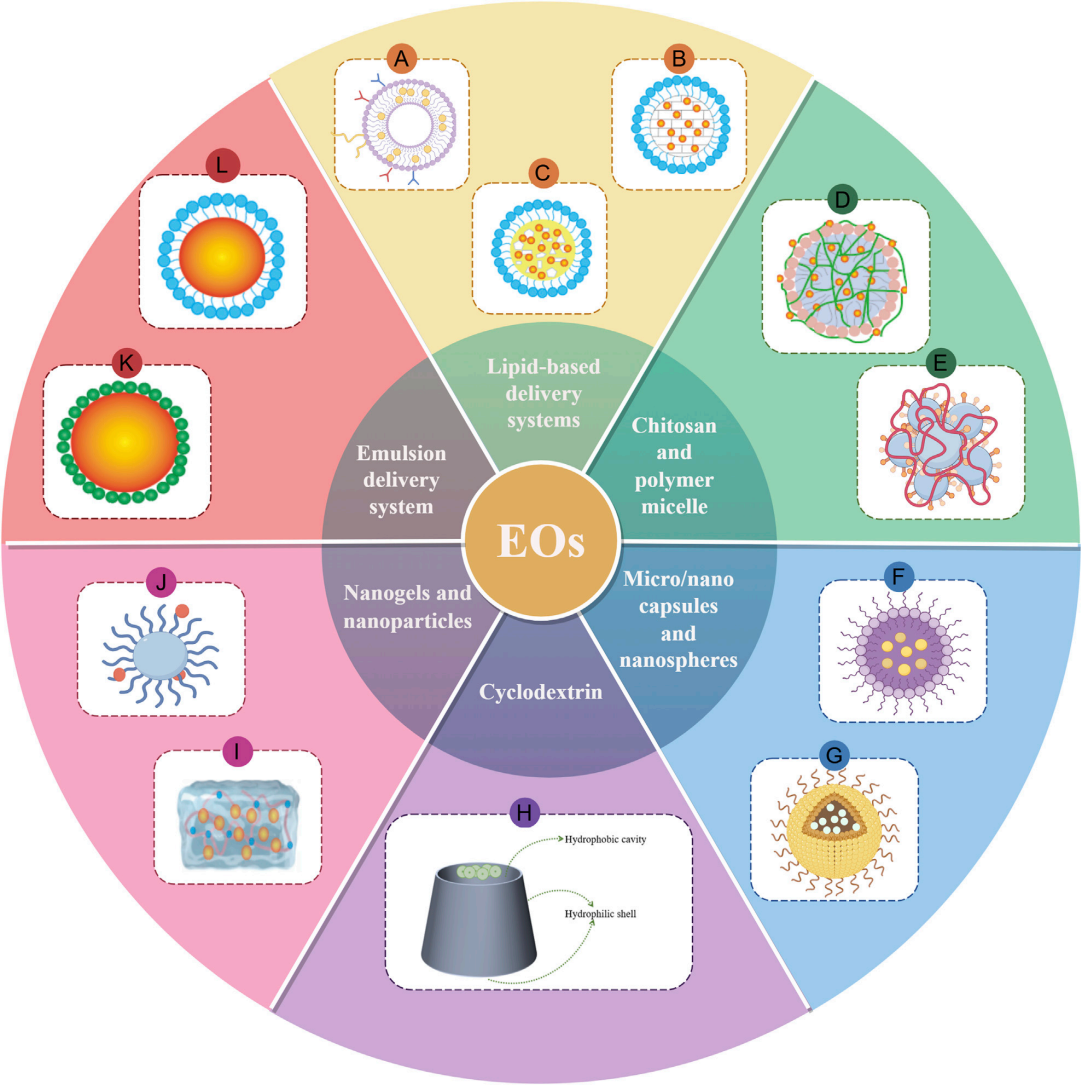
Summary of Different drug delivery system for EOs. Note: A, liposome; B, solid lipid nanoparticles; C, nanostructured lipid carrier; D, chitosan; E, polymer micelle; F, micro/nano capsules; G, nanospheres; H, cyclodextrin; I, nanogel; J, nanoparticles; K, Pickering emulsion; L, nano/micron emulsion. (By Figdraw).

In summary, the volatility, instability, poor solubility, and skin irritation of EOs lead to uneven curative effects and low bioavailability, making it impractical to use them alone. However, advances in natural plant research and the rapid development of drug delivery systems offer new solutions to these issues. A drug delivery system can effectively prevent the degradation of EOs by encapsulating them in or adsorbing them onto carriers, thereby improving their stability and bioavailability. These systems also allow controlled and targeted release, enabling EOs to act on the target area more accurately and effectively, reduce the toxicity, and further expand their application scenarios (Figure 6) (Chhetri et al., 2024; Lim et al., 2023; Ye et al., 2024).

### 5.1 Lipid-based delivery systems

#### 5.1.1 Liposomes (LS)

LS are vesicular structures that spontaneously assemble from one or more phospholipid bilayers surrounding an aqueous core, capable of encapsulating hydrophilic molecules within their internal aqueous phase and hydrophobic molecules within the phospholipid bilayers (Bedoya-Agudelo et al., 2024). LS, as the most mature nano-drug carrier, effectively addresses the instability and low solubility of EOs caused by external factors (such as reduced bioavailability) through the integration of EOs with LS technology (Hedayati et al., 2024). For instance, embedding *Origanum Vulgar* L. EO into LS can significantly enhance antioxidant activity and increase cytotoxicity against MCF-7 cancer cells (Kryeziu et al., 2022). Nanoliposomes (NLS) are LS with diameters typically under 200 nm, suited for high-precision targeting and controlled-release applications, such as local dermatological treatments and cancer therapy. Ellboudy et al. (2023) doped Cinnamon EO into an NLS formula to enhance its stability, thereby extending the release time and improving its antibacterial activity.

However, LS has limitations, such as poor repeatability, uneven particle size, and remarkable lipid oxidation. Consequently, the development of new carriers based on LS is on the rise. For instance, Baldim incorporated CD in LS to deliver Lippia sidoides Cham. and *Syzygium aromaticum* (L.) EO, finding that the EOs-in-CD-in-LS systems retained volatile metabolites and increased their physicochemical stability (Baldim et al., 2022). Similarly, Gan et al. (2024) enhanced NLS using the layer-by-layer electrostatic deposition method with chitosan and three anionic polymers (pectin, gum arabic, and carrageenan) as the first and second coating polymers, respectively, to encapsulate lemongrass EO, resulting in improved EO stability.

#### 5.1.2 Solid lipid nanoparticles (SLNs)

SLNs represent a novel colloidal drug delivery system that offers better stability compared to traditional LS. Comprised of three main metabolites—active metabolites (or drugs), solid lipids, and surfactants (Subroto et al., 2023)—SLNs utilize commonly biocompatible substances such as mono-, di-, and triglycerides, fatty acids, fatty alcohols, and waxes for the lipid core (Llaguno-Munive et al., 2024). The non-toxic and non-irritating nature of these lipids makes SLNs particularly suitable for injured or inflamed skin (Safta et al., 2024). Using a nanostructure design, EOs are encapsulated in SLNs as submicron capsules or nanoparticles, considerably enhancing the stability and bioavailability of the oils (AbouAitah and Lojkowski, 2022). For instance, Fuentes et al. (2024) developed an SLN delivery system based on *Mentha × piperita* L. EO and evaluated its thermal stability. The SLNs markedly reduced the evaporation loss of active metabolites in the EO, exhibited excellent thermal stability at 50°C and boosted the EO’s antibacterial properties. Additionally, SLNs can enhance the therapeutic efficacy of EOs. Due to their lipophilic nature and ease of penetration through the bacterial cell wall, SLNs can facilitate the delivery of Cinnamon EO across the cellular membrane, thereby enhancing its antibacterial activity against *E. coli* (Nemattalab et al., 2022). Similarly, Kotb et al. (2023) prepared *Boswellia sacra Flück.* EO into SLNs (FO-SLNs) *via* high-shear homogenization and assessed their anti-photoaging activity through *in vivo* experiments, demonstrating that FO-SLNs provided enhanced protection against UVB-induced epidermal thickening, dermal collagen degradation, and a decrease in inflammatory cell numbers compared to pure EO alone. While SLNs effectively protect EOs and improve their stability, they also present challenges such as complex production, high costs, and stability issues.

#### 5.1.3 Nanostructured Lipid Carriers (NLCs):

NLCs are second-generation lipid nanoparticles based on SLNs, formed from a mixture of solid lipids (cocoa, murumuru butter, and beeswax) and liquid lipids (olive oil or sesame oil). The presence of liquid lipids creates larger cavities within the solid lipids, ensuring a high drug-loading capacity and enhanced solubility of active metabolites. Importantly, the active metabolites rarely leak during storage (Cimino et al., 2021; Uchida et al., 2021). For instance, using NLCs to encapsulate Lippia sidoides Cham. and Caryophyllus aromaticus L. EOs results in a high encapsulation efficiency of the marker compound, ranging from 84.6% to 100%, significantly enhancing the therapeutic efficacy of EOs (Almeida et al., 2024). Pires et al. prepared seven types of NLCs; one formulation composed of Geranium EO and beeswax allowed storage for 210 days at 25°C. Meanwhile, NLCs prepared with encapsulated lemongrass EOs using murumuru butter, Cinnamon EOs with cocoa butter, Clove EOs, and Origanum vulgare L. EOs with beeswax demonstrated that encapsulation can improve the bioavailability, stability, and biocompatibility of EOs while reducing their photodegradation and toxicity (Pires et al., 2024).

In the EO-NLC system, liquid lipids may consist solely of EOs or be combined with additional liquid lipids to improve EO stability and enhance therapeutic efficacy. For instance, Santos Pimentel et al. (2024) prepared NLCs using Cinnamon, sage, and thyme as liquid matrices, combined with other solid matrices and surfactants. Experimental results demonstrated improved stability of all developed EO-based NLC formulations, while bioactivity remained unchanged. Satrialdi et al. (2024) prepared a Clove EO-loaded NLC (COE-NLC) via an emulsification-ultrasonication process, achieving an encapsulation efficiency of 97% and good stability. Furthermore, compared to free COE, COE-NLC exhibited enhanced free radical scavenging activity and effectively protected fibroblast cells from oxidative stress. The capacity of NLCs for EO loading is greater than that of LS and SLNs, although the use of surfactants in the preparation process is inevitable.

### 5.2 Emulsion delivery system

#### 5.2.1 Microemulsion (ME)

ME is a clear, uniform liquid mixture composed of a co-surfactant, water phase, oil phase, and surfactant. MEs can be categorized as O/W, W/O, or bicontinuous types. The nanoscale droplets created by surfactants in ME weaken the skin’s barrier function, making it suitable for transdermal drug delivery. The oil phase can improve drug absorption efficiency at the site of action, and using plant EOs, which dissolve the stratum corneum, as the oil phase further enhances ME permeability (Chen et al., 2024; Thakur et al., 2021). Wang et al. synthesized a ME of Matricaria recutita L. EO (MRME) using the phase inversion emulsification method and examined its anti-inflammatory and eczema-treating properties. Their findings indicated that MRME improved EO stability, lowered irritancy, and maintained anti-inflammatory and eczema-treating effectiveness (Wang et al., 2024a). Meanwhile, an ME loaded with Torreya grandis Fortune ex Lindl. EO (TaEO) showed higher stability and enhanced biological activity than pure TaEO (Wang et al., 2023a). Encapsulating Lavender, Basil, and Clove EOs using an ME, antibacterial experiments demonstrated more effective activity against *S. aureus* and *E. coli* at lower concentrations than those of pure EOs (Manzoor et al., 2023). Although ME significantly improves the efficacy of EOs, it has certain limitations, such as low encapsulation efficiency, poor stability, the use of surfactants during preparation, and residual organic solvents.

#### 5.2.2 Nanoemulsion (NE)

NEs are emulsion or colloidal dispersion systems composed of two immiscible liquids formed by surfactants, oil, and water, resulting in oil-in-water (O/W) or water-in-oil (W/O) types. Due to their high stability and greater resistance to environmental factors, NEs have found increased application in the food, cosmetics, and pharmaceutical industries. They enhance the stability, solubility, and bioavailability of active metabolites while producing a sustained-release effect (Huang et al., 2024a; Pandey et al., 2023; Shahabi et al., 2024; Sharma et al., 2024b). Sun et al. prepared an NE of Litsea cubeba (Lour.) Pers. EO (LEON) using ultrasonic emulsification; experimental data indicated that LEON significantly enhances the antibacterial efficacy and antioxidant capacity of the EO (Sun et al., 2024). Moreover, NE can also improve the antibacterial effect of EOs. For example, the NE of Callistemon citrinus (Curtis) Skeels EO exhibits 15 times the antibacterial activity against *E. coli* compared to pure EO and remains stable for up to 6 months (Haghbayan et al., 2024). Ling et al. prepared traditional emulsions (BDT) and NE (Bneo) of *Illicium verum* Hook. f. EO using ultrasonic methods; antibacterial results showed that the antifungal activity of Bneo against *Fusarium proliferatum* was 5.8 times higher than that of BDT, with good stability (Ling et al., 2024). However, the process inevitably requires surfactants and faces challenges related to thermodynamic instability and high production costs.

#### 5.2.3 Pickering Emulsion (PE)

Compared to emulsions traditionally stabilized with surfactants, PEs utilize solid particles to stabilize oil–water mixtures, including both O/W and W/O types. The stabilizing principle involves creating a solid barrier around the oil droplets, effectively isolating the oil phase from the water phase. The particles used as stabilizers are diverse, encompassing materials such as chitosan, starch, and cellulose. Owing to their low emulsifier dosage, ease of operation, strong biocompatibility, and high safety, they are increasingly favored. PE serves as an excellent carrier for EOs by reducing their sensitivity to evaporation and oxidation, thereby improving stability (Apostolidis et al., 2023; Cahyana et al., 2022; Pandita et al., 2024; Zhang et al., 2022b). For instance, a PE stabilized by modified cinnabar can enhance the thermal stability and *in vitro* dissolution rate of *Acorus tatarinowii* Schott EOs (Ru et al., 2024). PE can also carry lysozyme and tea tree EO, maintaining lysozyme activity while alleviating the volatilization of tea tree EO. Furthermore, the synergistic effect of the two metabolites exhibited a strong bactericidal effect against drug-resistant bacteria *in vitro* (Yao et al., 2024). Additionally, PE can improve the bioavailability of EOs, enhancing therapeutic efficacy and enabling sustained release. The PE of Cinnamon EOs demonstrates stronger antibacterial and antioxidant properties than pure EOs (Ly et al., 2024).

#### 5.2.4 Self-emulsifying drug delivery systems (SEDDS)

SEDDS are homogeneous mixtures of oil, surfactant, and co-surfactant. When in contact with an aqueous medium and subjected to gentle stirring, they spontaneously form an emulsion (AboulFotouh et al., 2017). Moreover, SEDDS avoid steps such as heating or solvent evaporation that may damage EO active metabolites or cause volatile losses, thereby overcoming issues like poor solubility, limited absorption, and inadequate stability (Liu et al., 2022a). For example, Chaisri prepared an SEDDS using EOs from *Cymbopogon citratus* (DC.) Stapf and lemongrass, finding that its stability was superior to that of the crude oils during both long-term storage and accelerated conditions (Chaisri et al., 2024). However, droplets formed by SEDDS for lipophilic, poorly water-soluble drugs may not release the active metabolites at all (Malkawi et al., 2020). An improved version, the Self-Nanoemulsified Delivery System (SNEDDS), features smaller emulsion droplets and generally better emulsifying properties, enabling the rapid formation of stable, consistent emulsions. When combined with other medications, Mentha × piperita L. and Lavender EOs can be encapsulated and released at the absorption site without interference from the surrounding environment (Ali et al., 2022; Alissa et al., 2023). Nonetheless, research in this direction remains limited.

### 5.3 Microcapsules and nanocapsules

#### 5.3.1 Microcapsules (MCs)

Introduced in the 1950s, MC technology involves encasing microscopic particles or droplets in polymer materials to create a core–shell structure. The outer layer protects the core (EOs) from harsh external conditions by efficiently blocking light, oxygen, and water. By precisely controlling the core material’s release, MCs mask its original odor and color while optimizing properties such as dispersion and solubility (Ma et al., 2024; Xiao et al., 2022). For example, Zhu et al. prepared MCs containing tea tree EO, which retained up to 87.1% of their content after high-temperature treatment for 70 min. Additionally, MCs enhance the inhibitory effect of EOs against *S. aureus* and *E. coli* (Zhu et al., 2024). While providing sustained release, MCs encapsulating Clove EO also boost the oil’s antioxidant properties (Li et al., 2024c). MCs containing lime peel EOs demonstrate good thermal stability, withstanding temperatures up to 122°C and maintaining an oil content of 38% over 4 weeks at room temperature (Indriyani et al., 2024). Composite MCs containing tea tree EO were fabricated using gelatin, Arabic gum, and n-butyl cyanoacrylate as wall materials via *in-situ* polymerization combined with composite solidification. Test results indicate that these capsules exhibit exceptional stability in osmotic environments and offer effective slow release and antioxidant capabilities under typical skin conditions (Yang et al., 2024b). However, MC preparation requires the use of surfactants.

#### 5.3.2 Nanocapsules (NCs)

NCs, which have a smaller diameter than that of MCs, consist of a core material designed to encapsulate lipophilic chemicals, enclosed within a polymer shell. This design considerably enhances the functionality of EOs by providing exceptional stability and controlled-release characteristics (Zhang et al., 2024b). Due to their nanoscale size, NCs can maintain prolonged contact with the skin, offering enhanced residence time. For example, NCs loaded with EO can completely inhibit *Pseudomonas acnes* and scavenge ROS, thereby protecting human skin cells and demonstrating strong skin permeability (Ivanova et al., 2023). Compared to free EOs, NCs containing EOs from *Rosmarinus officinalis* L. and *Lavandula dentata* L. improve deposition in the stratum corneum (Silva-Flores et al., 2023). Additionally, studies encapsulating basil EOs in NCs have shown that the NCs effectively mask the EO odor while preserving their antibacterial and antioxidant properties (Gorzin et al., 2024).

However, NCs require stringent storage conditions and are prone to leakage. Conversely, lipid NCs (LNCs) represent a monodisperse system capable of efficiently encapsulating hydrophobic active metabolites and achieving sustained drug release. For instance, combining Lavender oil with apocynin in LNCs enhanced targeting efficiency and prolonged residence time (Youssef et al., 2024). These LNCs also display superior permeability properties. The resulting LO-LNCs have improved the penetration of ASP through rat skin by encapsulating Lavender oil within LNCs. *In vivo* pharmacokinetic studies have shown that transdermal administration of LO-LNCs can quadruple the maximum plasma concentration of ASP, increase its bioavailability by up to 52%, and offer sustained release for up to 3 days compared to oral suspensions (El-Tokhy et al., 2023).

### 5.4 Polymer and gel delivery systems

#### 5.4.1 Polymeric Micelles (PMs)

PMs are self-assembled from amphiphilic copolymers, featuring a hydrophobic core for drug loading and a hydrophilic shell for drug absorption. As a delivery system for active molecules, PMs are easy to prepare, have a high drug-loading capacity, and their surfaces can be functionalized to achieve targeted delivery or extended circulation (Lodovichi et al., 2022). However, in recent years, there have been few studies on the encapsulation of EOs using PMs alone, with most efforts combining them with other carriers. For instance, *Origanum vulgare* L. Eos were first encapsulated into PMs and then incorporated into a binary hydrogel based on a Pluronic F127/L31 block-copolymer mixture. The pH compatibility of the EO-loaded poloxamer binary hydrogel with skin was investigated, and it exhibited sufficient spreadability and consistency. Furthermore, the formulation lowered the generation of inflammatory cytokines and limited the migration and proliferation of HaCaT cells (Bora et al., 2022). Most PM delivery systems focus on a single component rather than a complex EO, and future research should concentrate on loading EO mixtures.

#### 5.4.2 Nanogels (NGs)

NGs are nanoscale, three-dimensional network structures with a particle size range of 20–200 nm. They encapsulate active metabolites through self-assembly or crosslinking. NGs have broad application prospects in local administration due to their ease of use, high loading capacity, and excellent physical and chemical stability. Compared to LSs and NEs, NGs exhibit greater physical stability (Maurya et al., 2024; Zarenezhad et al., 2021). In a comparison between an NG and an NE-containing *Cuminum cyminum* L. EO, the IC50 value of the NG against A-375 human melanoma cells was lower than that of the NE. Furthermore, the inhibition rates of 5,000 μg/mL NG and NE against *Pseudomonas aeruginosa* and *S. aureus* were 100% and 80%, respectively (Ranjbar et al., 2023). Chitosan is often used as the cross-linking polymer in NGs. The EO of *Zataria multiflora* Boiss. And nisin were co-encapsulated in chitosan NGs to extend the shelf life of cheese. Experimental results demonstrated that within 60 days of storage, the chitosan NG slowed pH changes in the cheese, and no coliforms were detected, indicating sustained EO release (Hosseini, et al., 2024). Moreover, NGs can reduce the EO-induced irritancy caused by EOs. For instance, NGs containing *Lippia sidoides* Cham. EOsEO did not exhibit acute skin irritation in a rat excisional wound healing model and significantly reduced the final wound area (Pires Rodrigues de Almeida Ribeiro et al., 2024). In addressing multidrug-resistant strains, where effective treatments remain limited, Aldawsari et al. successfully encapsulated lemongrass EO using nanoparticles formulated with PVA and PLGA. This formulation maintained EO stability and bioactivity while enabling precise delivery to the target site, thereby enhancing LGO effectiveness against antibiotic-resistant *P. aeruginosa* (Aldawsari et al., 2023).

#### 5.4.3 Hydrogels

Hydrogels are hydrophilic, three-dimensional network gels capable of retaining large amounts of water and exhibiting excellent biocompatibility with bodily fluids. They can load active metabolites and release them in a controlled manner, making them suitable for the prolonged delivery of non-toxic, environmentally friendly bioactive substances. Additionally, they exhibit good air permeability and are used in wound healing (Alven et al., 2022; Elshahawy et al., 2024). Patchouli EO (PEO) shows limited absorption and low stability; therefore, a PEO-NE was developed and incorporated into a hydrogel composed of ROS/thermo dual-sensitive *Bletilla striata* polysaccharides. When administered rectally, PEONE-RTH adhered to the inflammatory site for an extended period and enhanced the antioxidant and repair activities of the EO (Huang et al., 2024b). As an external wound adhesive, hydrogels can load and promote the sustained release of EOs at injury sites, thereby improving bioavailability. For example, *R. officinalis* L., *Curcuma longa* L., and *Thuja occidentalis* L. EOs were loaded into a CCFG-CA hydrogel film; *in vitro* studies confirmed the film’s biocompatibility, antioxidant, and antimicrobial properties. Furthermore, *in vivo* wound healing studies showed that 14% of wounds healed and re-epithelialized within 99 days, with the hydrogel degradation time extended to 15 days (Tanwar et al., 2024).

The combined use of carriers can further reduce EO volatility and improve bioavailability. For example, Eucalyptus EOs in an NE prepared by physical crosslinking, when incorporated into a hydrogel, reduced the bacterial load of wounds and significantly downregulated inflammatory factor expression *in vivo* (Cai et al., 2023). In another example, a hydrogel containing an ME of *Alpinia officinarum* rhizome EO was developed for local application; the hydrogel maintained good physical stability after heating and cooling cycles at 4°C and during long-term storage (3 months) (Chittasupho et al., 2022).

### 5.5 Other delivery systems

#### 5.5.1 Chitosan (CS)

CS, a polysaccharide derived from the deacetylation of chitin, is a versatile and environmentally friendly biopolymer. Besides exhibiting antibacterial, antioxidant, and antitumor properties, CS is widely used due to its biodegradability, biocompatibility, and non-toxic characteristics. In combination with EOs, the bioactivity of CS can be further enhanced (Abenaim and Conti, 2023; Ding et al., 2022; Liu et al., 2024f). For instance, the CS-thyme EO combination retained an antiseptic effect after being stored for 12 days at low temperatures (Liu et al., 2024d). Rosmarinus officinalis L. EO exhibited high physical and chemical stability in CS and showed the highest inhibitory activity against *B. subtilis*, *E. coli,* and free radicals as measured by ABTS and DPPH assays (Akhter et al., 2024). CS particles loaded with geranium and lemongrass EO showed superior activity, better than that of pure CS or EO, by reducing the mixed biofilm of *C. albicans* and *Streptococcus mutans* on a glass slide and lowering the toxicity of EOs to RAW 264.7 cells (Garcia et al., 2023).

CS nanoparticles (CSNPs) are nano-sized particles derived from CS. By encapsulating Piper betle L. EO in CSNPs, the permeability of the EO can be improved, showing better solubility and efficacy than free EO (Muthusamy et al., 2024). In another study, SLNs and CSNPs were used as carriers to load tea tree EO. The MICs of EO-CS, EO-SLN, and pure EO against *S. aureus* and *P. aeruginosa* were 35 and 45, 130 and 170 μg/mL, and 380 and 410 μg/mL, respectively, with EO-CS exhibiting a considerably high antibacterial effect (Vase et al., 2023). While various EOs have been encapsulated by CS, limitations such as mechanical strength and water solubility mean that using CS as a sole carrier may not fulfill the requirements of some applications. Consequently, it is often necessary to compound or blend CS with other carriers, such as starch, cellulose, or protein, which can increase production costs.

#### 5.5.2 Cyclodextrin (CD)

CD is a water-soluble, biodegradable cyclic oligosaccharide that produces a mixture of α-CD, β-CD, and γ-CD composed of 6, 7, and 8 glucopyranose units, respectively. The hydrophobic cavity of CD can form complexes with various water-insoluble substances (such as EOs), thereby enhancing its stability and solubility and delaying volatilization (Ma et al., 2023; Paiva-Santos et al., 2022; Rodrigues Arruda et al., 2022). Among these, β-CD, containing seven sugar units, is widely used owing to its moderate size, which allows it to encapsulate most EOs with low sensitivity and irritation to the skin (Wu et al., 2024). β-CD active bacterial nanocellulose (BNC) nanopapers containing Salvia officinalis L. EO (SEO) were prepared. Experimental results demonstrated that adding SEO-βCD complexes improved the thermal properties of the BNC nanopapers, enhanced the antibacterial effect against *L. monocytogenes*, and increased antioxidant capacity (Mohammadzadeh et al., 2024). Ben et al. prepared β-CD/Eucalyptus globulus Labill. EO (EGEO) inclusion complexes, and the results showed that compared to the free EO (8.38 ± 1.95 mg/g), the β-CD/EGEO inclusion complexes exhibited an LC_50_ of 5.03 ± 1.16 mg/g against *Ephestia kuehniella* larvae, indicating enhanced EO activity (Ben Amara et al., 2024). Preparing an inclusion compound of fennel EO and hydroxypropyl-β-CD regulated EO release extended its release duration and improved its stability under varying temperature and relative humidity conditions (Song et al., 2024). Through β-CD encapsulation, the release of Cinnamon EO was delayed to about 90 h, achieving a high growth inhibition rate of almost 100% for *E. coli* and *S. aureus* (Li et al., 2023a).

#### 5.5.3 Mesoporous Silica Nanoparticles (MSNPs)

MSNPs possess a high surface area and well-ordered protective pore structure, exhibiting excellent chemical stability, biocompatibility, and biodegradability. Embedding EO into MSNP can improve its stability and water solubility while achieving more sustained therapeutic efficacy through controlled release. After 2 months, the retention rate of the encapsulated compound reaches 50%. The synthesis method of MSNPs is simple and economical, making it suitable for large-scale industrial production (Gulin-Sarfraz et al., 2022; Lai et al., 2023; Weisany et al., 2024). The encapsulation of Lippia graveolens Kunth EO in MSNPs preserved its antioxidant and antibacterial properties, exhibiting a release rate of up to 42 days and inhibiting the growth of pathogenic and spoilage microorganisms (Matadamas-Ortiz et al., 2022). The surface modification of mesoporous silica particles (MSPs) reduces the hydrophobicity of EO, improves its solubility in the water phase, and enables it to penetrate into fungal cells, destroying the cell membrane and eventually causing cell death. Chakroun et al. encapsulated Ammoides pusilla (Brot.) Breistr. EO (AP-EO) into MSPs using an impregnation method and coated the surface with CS. In agar medium contact tests, the antifungal activity of AP-EO in MSPs without CS increased threefold. However, the CS coating slowed EO release. Therefore, using multiple carriers can further improve the stability of EO, achieve slow release, and enhance therapeutic effects (Chakroun et al., 2023).

To sum up, combining EO with modern drug delivery systems can minimize their defects and fully exploit their therapeutic potential. This section briefly summarizes modern drug delivery systems for encapsulating EOs. However, it is worth noting that EO delivery systems face some limitations. Therefore, researchers are urged to develop new strategies to overcome these shortcomings and optimize the effective delivery of EOs. For instance, Carneiro et al. utilized HPH technology to prepare NEs and NLCs containing Piper aduncum L. Eos, which were then thickened with hydrogel, enhancing both the viscosity and skin adherence of the nanoformulations (Carneiro et al., 2022). Chittasupho et al. successfully applied Kaempferia galanga L. EOs in ME to hydrogel, showing good physical stability and potential as a local sunscreen preparation (Chittasupho et al., 2022). For another example, garlic EO is limited in its application because of its irritation, poor water solubility, and low bioavailability. Therefore, Zhang et al. combined garlic EO with β-CD inclusion liposome to prepare a double-layer delivery system, which not only masked the smell of EO but also significantly improved its embedding efficiency and reduced the release rate (Zhang et al., 2024c). Elsewedy et al. integrated NE-containing fusidic acid and *Cinnamomum cassia* (L.) J. Presl EO into a hydrogel matrix, which prolonged the *in vitro* release time and enhanced the stability and antibacterial effects (Elsewedy et al., 2024). New carriers or materials have also been developed and utilized. For instance, a novel delivery system of nanoemulgel, combining NE with a gel matrix, merges the small particle size and high stability of NE with the controlled release and good skin adhesion of the gel. This includes the Zanthoxylum armatum DC. and *R. officinalis* L. EO nanoemulgel (Noor et al., 2023) and the nanoemulgel of Pituranthos tortuosus EO (Bahloul et al., 2024). Moreover, Fan et al. developed PE co-loaded with tannic acid and Cinnamomum cassia (L.) J. Presl EO based on zein and tannic acid complexes through interfacial engineering. This improved the stability and controlled-release performance of EOs compared to using PE alone (Fan et al., 2024). ME is widely used in the field of EO encapsulation; however, a common limitation is the use of potentially toxic surfactants and co-surfactants. Shen et al. used rhamnolipids instead of surfactants with rose and eucalyptus EOs to formulate MEs. Compared to regular emulsions, the EO-ME exhibited better slow-release behavior. The EO-ME significantly lowered the MIC against *S. aureus* (39 mg/L, compared to 1,250 mg/L with EO alone), demonstrating stable performance during storage (Shen et al., 2024a). Furthermore, Cheng and colleagues developed carrier-free nanodelivery systems characterized by self-assembly from active metabolites without surfactants or carriers (Cheng et al., 2024).

## 6 The drug delivery system enhances the physical and chemical properties of essential oil

One of the primary challenges in broadening the application of EOs is enhancing their bioavailability. Over the past decade, the use of drug delivery systems for encapsulating EOs has gradually increased. Numerous studies show that these systems can improve the stability and targeting of EOs, reduce their toxicity, and facilitate slow release, thus effectively improving EO bioavailability.

### 6.1 Sustained release of essential oil

Maintaining drugs within the therapeutic concentration range is crucial for chronic diseases requiring long-term treatment. However, frequent drug administration not only inconveniences patients but can also lead to drug resistance. Research has shown that drug delivery systems can maintain the slow release of EOs, achieving continuous treatment effects. For instance, Long et al. conducted an *in vitro* release study on Ligusticum striatum DC. EO (CXEO)-LS, which indicated a gradual increase in cumulative release within 48–72 h and demonstrated good storage stability at 4°C for at least 25 days (Long et al., 2023). Additionally, researchers have developed a photoresponsive LS for the controlled release of Alpinia galanga EO by utilizing the photoreactivity of Pheophorbide-a. This system has shown strong sustained-release effects and can be stored for up to 28 days at 4°C (Ge et al., 2023). Shen et al. developed PEs using potato protein-CS composite nanoparticles (PCCNs) for encapsulating *Zanthoxylum bungeanum* Maxim. EO (ZBEO). Confocal laser scanning microscopy revealed that PCCNs adsorbed onto the EO surface to form a dense interfacial layer, significantly enhancing the stability of ZBEO-PEs and enabling sustained EO release (Shen et al., 2024b). Alam et al. encapsulated fennel EO (FEO) into poly (lactic-co-glycolic acid) nanoparticles (FEO–PLGANPs) and incorporated them into NGs. In an *in vitro* drug release study, over 60% of the pure EO was released within 120 min, whereas the release amounts for FEO–PLGANPs NGs and FEO–PLGANPs were 31.43% and 26.76%, respectively, indicating that encapsulation within nanoparticles and NGs significantly delayed the drug release rate (Alam et al., 2022). Thus, EO drug delivery systems provide a viable solution for maintaining long-term therapeutic concentrations and improving patient convenience.

### 6.2 Enhancing the stability of essential oil

The stability of EOs is often disturbed by external environmental factors. Encapsulating EOs within drug delivery systems can effectively isolate them from these external conditions, thereby improving their physical and chemical stability. Wei et al. developed thyme EO-ME (TEO-M) and incorporated it into pullulan-sodium alginate (PS) films, finding that TEO-M endowed the PS films with antioxidant and UV-blocking properties, which remained stable for 10 days at 4°C (Wei et al., 2023). The NE of *Calotropis gigantea* EO remained stable after being stored at various temperatures for 50 days (Sharma, et al. 2024a). Cinnamon EO-MC prepared using the coagulation method extended its shelf life beyond 10 days when applied to kraft paper wrapped around citrus (Liu, et al. 2024e). Additionally, combining two carrier systems can further stabilize EOs. Xing et al. prepared NEs encapsulating Cinnamon EOs using hydroxypropyl-β-CD/lauroyl arginate inclusion complexes and found that under 100 MPa pressure and after seven cycles, the NEs exhibited excellent storage stability and thermal stability (Xing et al., 2024).

### 6.3 Enhancing the targeting function of essential oil

Drug delivery systems for EOs can be engineered to incorporate specific ligands on the carrier surface, thereby enabling active targeting of EOs to lesions through receptor-ligand interactions and enhancing therapeutic efficacy. For instance, Mahmoud et al. encapsulated thyme EO within O-quaternized, ultrasound-mediated deacetylated chitosan NCs, which not only exhibited excellent stability and release properties but also demonstrated selective targeting toward the SARS-CoV-2 virus (Mahmoud et al., 2024). Researchers have developed a novel lipid nanocapsule that encapsulates apocynin, is coated with lactoferrin, and incorporates Lavender EO as a bioactive additive. This system effectively targets the brain, where Lavender EO and apocynin work synergistically to alleviate seizures induced by pentylenetetrazol (Youssef et al., 2024). Additionally, Chen et al. prepared LS of Clove EO by embedding casein using the freeze-thaw method and then coupled *Campylobacter* jejuni antibodies to the LS surface *via* the post-insertion method, producing protease-activated, antibacterial LS with bacterial targeting capabilities (Chen et al., 2023b).

### 6.4 Reducing the toxic effects of essential oil

EOs are rarely applied directly to the skin without dilution because they may cause severe irritation. Therefore, the EU Cosmetics Regulation (EC) No 1223/2009 restricts the use of certain EO metabolites (such as coumarin and camphor) and mandates that known allergens (such as linalool and limonene) be labeled when their concentrations exceed specified limits. Additionally, products containing EOs must undergo safety assessments before being marketed. In 2001, the Scientific Committee on Cosmetic Products stipulated that the total concentration of furocoumarins in cosmetics must not exceed 1 ppm (Kejlová et al., 2010). In practical applications, EOs are typically diluted with carrier oils (such as vegetable oils, fixed oils, or fats), cream bases, or gels, with concentrations generally ranging from 2% to 5%. For more irritating EOs, the maximum dilution concentration should be reduced to 0.5% to ensure safe use (Dontje et al., 2024). Recent studies have shown that combining EOs with drug delivery systems can effectively reduce their toxicity and skin irritation. For instance, when using NLC to load Red Sacaca EO (NLC-RSO), the cytotoxicity of NLC-RSO was significantly lower than that of free RSO, indicating a cytoprotective effect of NLC on EO delivery (Chura et al., 2023). When applied topically, Cinnamon EO may cause skin irritation and phototoxicity. However, Essid et al. reduced its cytotoxicity by encapsulating Cinnamon EO in CSNPs, achieving a fourfold decrease compared to pure Cinnamon EO (Essid et al., 2023). Emtiazi et al. encapsulated *Achillea millefolium* EO using NLS/nano-niosomes, noting that the encapsulated EO exhibited lower toxicity than that exhibited by its non-encapsulated form (Emtiazi et al., 2022). Alam et al. encapsulated FEO into FEO–PLGANPs and further integrated them into NGs. *In vitro* cell viability assays using the normal cell line L929 demonstrated that both FEO–PLGANPs and FEO–PLGANPs NGs exhibited low toxicity and excellent biocompatibility, making them highly suitable for topical applications without irritation (Alam et al., 2022).

## 7 Effects of plant essential oil-based skin care on the nervous system

**FIGURE 7:**
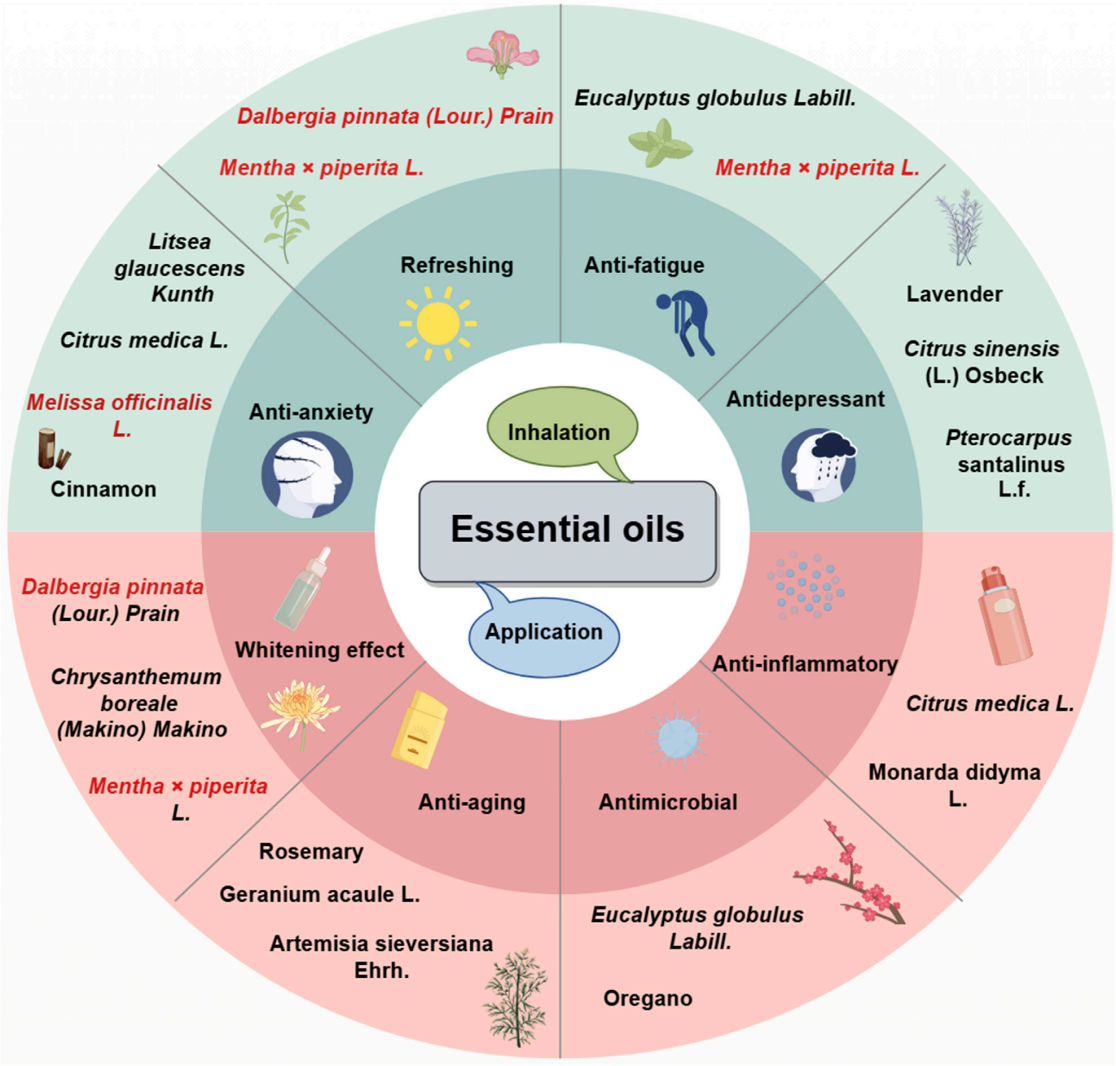
EOs with positive effects caused by inhalation. Note: Layer 1: EOs can be administered in two primary ways: inhalation and daubing. Layer 2: Inhalation, represented by green, is associated with benefits such as anti-anxiety, anti-depression, anti-fatigue, and a refreshing effect, while topical application, denoted by pink, delivers skin care benefits, including whitening, anti-aging, anti-bacterial, and anti-inflammatory properties. Layer 3: List the EOs related to the second layer, respectively. Some EOs (marked in red font) can be inhaled and smeared at the same time, indicating that EOs can nourish and repair the skin and also have a positive impact on the mental health of users. (By Figdraw).

When individuals experience mental stress and fatigue, neuromodulators are released from skin nerve fibers, which can impair skin function and reduce its resilience to environmental stressors. Due to their rich active metabolites, EO-based skincare products not only nourish and repair the skin but also positively impact human psychology. For instance, a study showed that inhalation of Vetiver EOs dramatically altered gene expression in the lateral region of the central amygdala, displaying anti-anxiety characteristics (Saiyudthong et al., 2015). Díaz-Cantón et al. demonstrated that inhaling the EO of Litsea glaucescens Kunth increases brain-derived neurotrophic factor levels in the brains of mice, thereby exhibiting significant anxiolytic effects (Díaz-Cantón et al., 2024). Li et al., using the Forced Swim Test, Sucrose Preference Test, and Open Field Test, showed that inhaling Lavender EO significantly alleviated depressive-like behaviors in rats undergoing alcohol withdrawal (Li et al., 2024b). Sgoifo et al. explored the stress resistance properties of EO-rich skincare through self-administration experiments; their findings indicate that this approach reduces stress and promotes psychological recovery by lowering performance anxiety, state anxiety, and nonverbal behavioral patterns (Sgoifo et al., 2021). Therefore, incorporating plant EOs into skincare leverages the dual effects of the olfactory and epidermal systems to nourish and repair the skin, enhance the user’s emotional state, and regulate physiological functions simultaneously. Figure 7 summarizes EOs used to improve human negative emotions, and some EOs can produce dual pharmacological effects, both through inhalation and topical application, which can provide far-reaching effects when added to skin care products.

## 8 Future perspectives and conclusion

As interest in natural and healthy skincare methods grows, EOs are increasingly utilized in skincare for their unique efficacy and natural purity. Projections for 2026 estimate that the global EO market will reach $16 billion (Tsitlakidou et al., 2023). The skin-whitening, UV resistance, antioxidative, antibacterial, and anti-inflammatory properties of EOs are well recognized. However, several challenges remain. First, the complexity of EO metabolites makes them susceptible to factors such as the plant’s growth stage, the specific parts used for extraction (Al-Snafi et al., 2024), and the extraction techniques adopted (Park et al., 2024). All these factors can significantly affect EO quality and the concentration of active metabolites. Additionally, isolating these metabolites from complex mixtures is challenging, and their release mechanisms are not yet fully elucidated. Second, direct application of EOs on the skin may cause adverse reactions, including phototoxicity, eye irritation, and skin allergies. Therefore, encapsulation represents a crucial strategy for broadening the safe application of EOs. Third, as the EO market continues to expand, counterfeit products and consumer misinformation are becoming more prevalent, leading to suboptimal usage outcomes.

Given the high sensitivity of EOs to environmental conditions, their storage requires special considerations. Employing various carriers has proven effective in mitigating the adverse effects of changes in O_2_, temperature, humidity, and pH, thereby ensuring the quality and stability of EOs. This paper briefly discusses several important carrier delivery systems and related preparation technologies. Combining EOs with these systems enhances their dispersion, stability, and release kinetics, improving overall efficacy compared to free EOs (Dupuis et al., 2022). However, several challenges persist. First, many existing EO delivery systems are complex to prepare, costly, and lack long-term stability. For instance, nano-lipid carriers may transform into a thermodynamically stable crystalline state during storage, reducing their drug-carrying capacity and potentially causing abrupt drug release (Pereira et al., 2018). Second, safety concerns arise because nanocarriers, while enhancing therapeutic effects by improving permeability, also increase the risk of cytotoxicity and potential harm to the nervous system. Moreover, the use of organic solvents or surfactants in nanopreparation processes may leave residues that adversely affect human health (Najahi-Missaoui et al., 2020). Third, current studies typically focus on encapsulating a single EO with one carrier; exploring the possibility of encapsulating multiple EOs within a single carrier presents significant research opportunities. Fourth, each delivery system has its limitations. Although combining two or more carriers can further enhance EO stability, cost considerations must also be taken into account.

In summary, to expand the application of EOs in the skincare field, it is essential to advance experimental research on transdermal drug delivery beyond *in vitro* studies and increase focus on both acute and chronic toxicity, including potential impacts on the liver, nervous system, and other organs following skin absorption. Further investigation is required to determine safe concentration ranges and ensure that experimental findings consistently translate into clinical therapeutic outcomes. In addition, standardizing extraction methods for various EOs, strengthening market regulation, and establishing clear laws and guidelines are necessary steps to safeguard consumers. Enhanced consumer education regarding the safe use of EOs and the identification of counterfeit products will also promote a healthier industry.

Concurrently, improving existing EO delivery systems by enhancing encapsulation efficiency and optimizing sustained-release performance to reduce toxicity and irritation while minimizing the use of harmful solvents is a key research priority. Ultimately, establishing a comprehensive safety evaluation system that spans from cell experiments and animal models to human trials is vital for fully assessing the potential risks of EOs. This comprehensive approach will drive their application in skincare, offering a safer, more effective, and environmentally friendly therapeutic solution.

## Glossary

**EOs**: Essential oils
**TYR**: tyrosinase
**DHI**: 5,6-dihydroxyindol; DOPAdihydroxyphenylalanine
**DHICA**: 5,6-dihydroxy indole-2-carboxylic acid
**DCT**: Dopachrome tautomerase
**TRP**: tyrosinase-related protein
**MITF**: microphthalmia-associated transcription factor
**MAPK**: Mitogen-Activated Protein Kinase
**IC50**: half-maximal inhibitory concentration
**TD50**: half-maximal toxicity dose
**μM**: micromolar
**ROS**: reactive oxygen species
**B16-F10**: malignant melanoma cell line of C57BL/6 J mice
**A375**: human melanoma cell line
**B16BL6**: melanoma-associated cell line
**α-MSH**: Alpha-Melanocyte-Stimulating Hormone
**B16**: melanoma-associated cell line
**UVR**: ultraviolet radiation
**LPO**: lipid peroxide
**USFA**: unsaturated fatty acids
**ROS**: reactive oxygen species
**CAT**: antioxidant enzyme catalase
**GST**: glutathione-S-transferase
**BHT**: 2, 6-di-tert-butyl-p-cresol
**FRAP**: ferric ion reducing antioxidant power
**DPPH**: 2, 2-diphenyl-1-picrylhydrazyl
**ABTS**: 2,2′-azino-bis(3-ethylbenzothiazoline-6-sulfonic acid
**EC50**: median effective concentration
**IL**: interleukin
**IL-6**: Interleukin-6
**IL-1β**: Interleukin-1β
**COX-2**: Cyclooxygenase-2
**PGE2**: Prostaglandin E2
**MMP-1 and MMP-3**: Matrix Metalloproteinases-1,-3
**TNF-α**: Tumor Necrosis Factor-α
**HaCaT**: human keratinocyte cell line
**HDFn**: human dermal fibroblast cells
**SPF**: sun protection factor
**SOD**: superoxide dismutase
**GSH-Px**: glutathione peroxidase
**MDA**: malondialdehyde
**MIC**: minimum inhibitory concentration
**MBC**: minimum bactericidal concentration
**DIZ**: diameter of inhibition zone
**TNF**: tumor necrosis factor
**LPS**: lipopolysaccharide
**DSS**: dextran sulfate sodium
**PGE**: prostaglandin E
**COX**: cyclooxygenase
**TLR**: toll-like receptor
**p-HBA**: methyl p-hydroxybenzoate
**BuP**: butyl p-hydroxybenzoate chlorobenzyl etherSBsodium benzoate
**ATP**: adenosine triphosphate
**ADP**: adenosine diphosphate
**G+**: Gram-positive bacteria
**G-**: Gram-negative bacteria
**ZOI**: zone of inhibition
**MIC**: minimum inhibitory concentration
**MBC**: minimum bactericidal concentration
***E. coli***: *Escherichia coli*
***B. subtilis***: *Bacillus subtilis*
***S. aureus***: *Staphylococcus aureus*
**C. albicans**: *Candida* albicans
***P. aeruginosa***: *Pseudomonas aeruginosa*
**B. bronchiseptica**: *Bordetella* bronchiseptica
**S epidermidis**: *Staphylococcus* epidermidis
***L. monocytogenes***: *Listeria* monocytogenes
***S. typhimurium***: *Salmonella typhimurium*
***S. typhi***: *Salmonella typhi*
**DIZ**: diameter of inhibition zone
**5-LOX**: Lipoxygenase
**NO**: nitric oxide
**iNOS**: nitric oxide synthase
**AA**: Arachidonic Acid
**LS**: Liposomes
**NLS**: Nanoliposomes
**SLNs**: Solid Lipid Nanoparticles
**NLCs**: Nanostructured Lipid Carriers
**ME**: Microemulsion
**NE**: Nanoemulsion
**PE**: Pickering Emulsion
**SEDDS**: Self-emulsifying drug delivery systems
**SNEDDS**: Self-Nanoemulsified Delivery System
**MCs**: Microcapsules
**NCs**: Nanocapsules
**PMs**: Polymeric Micelles
**NGs**: Nanogels
**CS**: Chitosan
**CD**: Cyclodextrin
**MSNPs**: Mesoporous Silica Nanoparticles.
